# A recombination between two Type 1 Porcine Reproductive and Respiratory Syndrome Virus (PRRSV‐1) vaccine strains has caused severe outbreaks in Danish pigs

**DOI:** 10.1111/tbed.13555

**Published:** 2020-05-25

**Authors:** Lise Kirstine Kvisgaard, Charlotte Sonne Kristensen, Pia Ryt‐Hansen, Kasper Pedersen, Tomasz Stadejek, Ramona Trebbien, Lars Ole Andresen, Lars Erik Larsen

**Affiliations:** ^1^ National Veterinary Institute Technical University of Denmark Lyngby Denmark; ^2^ SEGES Danish Pig Research Centre Aarhus Denmark; ^3^ Faculty of Veterinary Medicine Warsaw University of Life Sciences Warsaw Poland; ^4^ Statens Serum Institut Copenhagen Denmark; ^5^ Department of Health and Medical Sciences Institute for Veterinary and Animal Sciences University of Copenhagen Copenhagen Denmark

**Keywords:** porcine reproductive and respiratory syndrome virus, PRRSV, recombination, swine

## Abstract

Porcine reproductive and respiratory syndrome virus (PRRSV) is prevalent in Danish swine herds. In July 2019, PRRSV‐1 was detected in a PRRSV‐negative boar station and subsequently spread to more than 38 herds that had received semen from the boar station. Full genome sequencing revealed a sequence of 15.098 nucleotides. Phylogenetic analyses showed that the strain was a recombination between the Amervac strain (Unistrain PRRS vaccine; Hipra) and the 96V198 strain (Suvaxyn PRRS; Zoetis AH). The major parent was the 96V198 strain that spanned ORFs 1–2 and part of ORF 3 and the minor parent was the Amervac strain, which constituted the remaining part of the genome. The virus seems to be highly transmissible and has caused severe disease in infected herds despite a high level of genetic identity to the attenuated parent strains. The source of infection was presumable a neighbouring farm situated 5.8 km from the boar station.

## 1 INTRODUCTION

Porcine reproductive and respiratory syndrome virus (PRRSV) has since its first appearance in the beginning of the 1990s been one of the major health challenges in pig production. According to figures from the Danish SPF society, approximately 35% of the Danish pig herds are positive for antibodies to PRRSV, but the prevalence of serological‐positive herds is declining (www.spfsus.dk). Both PRRSV‐1 and PRRSV‐2 are prevalent in Danish herds, and some herds are infected by both species. There is a variety of PRRSV programs in place for the control of PRRSV in different herds, but in many herds, PRRSV is circulating in the nursery and among growers/fatteners. Mass sow vaccinations are used in many herds, whereas piglet vaccination is less commonly used. Modified live virus (MLV) vaccines are presently used in Denmark to control PRRSV. The MLV PRRSV‐1 vaccine Porcilis (MSD Animal Health) was introduced in the late 1990s and was, until 2017, the only PRRSV‐1 MLV vaccine used in Denmark. In December 2016, the vaccine Unistrain (Hipra, Spain) was launched, and in August 2018, the PRRSV‐1 MLV Suvaxyn PRRS (Zoetis Animal Health, USA) was introduced.

A limited surveillance of the genetic diversity is practised in Denmark, but the available data indicate that two major clades of PRRSV‐1 are co‐circulating (Kvisgaard, Hjulsager, Kristensen, Lauritsen, & Larsen, 2013). One of the clades shares a high level of genetic similarity to the Porcilis vaccine strain ‘DV’ and probably represents a group of field viruses originating from this vaccine strain. The strains clustering in the other clade are up to 12% different from the ‘Porcilis‐like’ viruses and include the first PRRSV‐1 strain isolated in Denmark in 1992 (Kvisgaard, Hjulsager, Kristensen, et al., 2013). All of these viruses belong to the PRRSV‐1, subtype 1, whereas PRRSV‐1 strains belonging to subtypes 2, 3 and 4 never have been detected in Denmark (Stadejek, Oleksiewicz, Potapchuk, Podgorska, & Podgórska, 2006; Stadejek et al., 2002, 2008).

Several breeding companies are supplying semen to Danish sow herds, the largest supplying semen to more than 860,000 of the Danish production sows. This breeding company consists of 14 boar stations, where four are defined as PRRSV seropositive and 10 are PRRSV seronegative. The negative boar stations recruit boars from PRRSV‐negative breeding herds, and the boars are subjected to 35–40 days of quarantine prior to introduction into the boar station. The PRRSV‐negative boar stations are surveyed for PRRSV antibodies by ELISA by bi‐monthly test of at least one boar per section. The last outbreak of PRRSV in a Danish negative boar station dates back to 2002.

In July 2019, PRRSV‐1 was detected in samples taken as part of the routine PRRSV surveillance in one of the PRRSV‐negative boar stations. The virus was shortly after detected in three nucleus herds and in at least 38 production herds that had received semen from this station. Tracking of the virus and extensive test of the connected herds are ongoing; however, the preliminary reports from the infected herds indicate that the virus may induce clinical signs similar to or even exceeding those normally observed in Danish herds infected with PRRSV‐1. The clinical signs include sustained reproductive failures and high piglet mortality. Despite that the outbreak is not fully investigated, we find it appropriate to submit this rapid outbreak report with focus on the genetic composition and the epidemiology of the new PRRSV strain with the primary aim to alert countries that import living pigs from Denmark.

## 2 MATERIALS AND METHODS

### 2.1 Herds and samples

The index case—the boar station—contained 261 boars that were divided into six sections. There were no restrictions of movement of people or equipment between the sections, so the station was considered to consist of a single epidemiological unit.

In total, 71 nucleus and multiplier herds and more than 650 production herds received semen from the boar station between 1 July 2019 and 24 July 2019.

Serum samples were collected from inseminated sows or from other sows present in the herds. Samples were also collected in some of the production herds, but the total number of tested production herds is not known.

The Suvaxyn PRRS (Zoetis AH) has only obtained a very limited marked share in Denmark; however, search in the database on medicine and vaccines used in Danish herds (VETSTAT) revealed that one herd located 5.8 km west of the boar station had used both vaccines in different age groups (sows and weaners) during 2019. Therefore, 50 blood samples were collected in this herd the 13th of August, subsequent to the outbreak at the boar station.

### 2.2 ELISA

The serum samples were tested for antibodies against PRRSV using a commercial available ELISA (IDEXX PRRS X3 Ab Test, The Netherlands).

### 2.3 RNA extraction and real‐time RT‐PCR assays

Total RNA was extracted from serum and lung tissue homogenate supernatant using the QIAGEN QIAcube extraction robot. Total RNA from serum was extracted from 140 µl aliquots with the QIAamp^®^ Viral RNA Mini Kit (QIAGEN) utilizing the protocol: ‘Purification of viral RNA from cell‐free body fluids’ and eluted in 60 µl. Lung tissue homogenate supernatant was prepared (Kvisgaard, Hjulsager, Fahnøe, et al., 2013) and total RNA was extracted from 200 µl aliquots with RNeasy^®^ Mini Kit (QIAGEN) utilizing the protocol: ‘Purification of total RNA from easy‐to‐lyse animal tissues and cells (large samples)’ and eluted in 60 µl. A known PRRSV‐positive sample and a negative control were included in each round of extraction. The RNA was stored at −80°C.

Total RNA was extracted manually from cell culture supernatants using the QIAamp^®^ Viral RNA Mini Kit (QIAGEN) following the manufactures guidelines. RNA was eluted in 60 µl and stored at −80°C.

To screen for PRRSV‐1 and PRRSV‐2, a multiplex dual‐labelled real‐time RT‐PCR assay ‘Kleiboeker mod‐1’ primers and probe targeting ORF6 and ORF7 was used (Wernike et al., 2012).

### 2.4 Conventional PCR and sequencing

cDNA was synthesized with a 1:1 mix of random hexamer and poly(T) primers and otherwise as previously described (Kvisgaard, Hjulsager, Fahnøe, et al., 2013).

Individually, amplifications of ORF2 through ORF7 were amplified with cDNA as template using the AccuPrime™ *Taq* DNA Polymerase High Fidelity kit (Invitrogen) following the guidelines from the supplier except that the amount of polymerase was increased to 0.5 µl per reaction. Purified PCR products were sequenced by Cycle Sequencing (Sanger, Nicklen, & Coulson, 1977) at LGC genomics GmbH (Berlin, Germany) with the PCR primers as sequencing primers.

For full genome sequencing, the PRRSV genome was amplified in four overlapping fragments with AccuPrime™ Taq DNA Polymerase High Fidelity kit (Invitrogen) with 1 µl of polymerase per reaction and elongation times of 60 s per kilobase.

The four fragments covering the complete PRRSV genome were pooled in an equimolar concentration and submitted for next generation sequencing (NGS) at Statens Serum Institut (SSI). The library and fragmentation were prepared from Nextera XT DNA Library Preparation Kit (Illumina), and DNA concentration was measured using Quant‐iT™ dsDNA High‐Sensitivity Assay Kit (Life Technologies). The sequencing was performed on the MiSeq platform (Illumina) with the Miseq Reagent Kit v2 (500 cycles).

All primers, annealing temperatures and elongation times for amplification of PCR products are listed in Table S1.

### 2.5 Assessment of sequences and phylogenetic analyses

Raw cycle sequencing data of individually ORFs were assembled and analyzed using the commercial software CLC main Workbench v. 8.1.2 (QIAGEN). Primer binding sequences were excluded to avoid bias from primer mismatch.

The quality of the NGS data was assessed by the FastQC applicant (v0.11.8). The trimming and assembly of NGS data were done with the commercial software CLC Genomics v11.0.1 (QIAGEN). The data were assembled by ‘assembly to a reference’ using the Amervac (GU067771) vaccine strain (Unistrain) as reference sequence, and the consensus sequence was extracted. Blastn analysis was performed against NCBI Genbank (https://www.ncbi.nlm.nih.gov) to determine the closest sequence match.

Phylogenetic trees representing ORFs1‐7, ORF2, ORF3, ORF4, ORF6 and ORF7 were created using the consensus sequence of the case virus isolated from the boar station (the ‘Horsens’ virus), and 92 PRRSV‐1 sequences available in GenBank. For the phylogenetic analysis of ORF5, all Danish viruses previously sequenced were also included, resulting in a total of 171 ORF5 sequences. Sequence alignments were performed with MUSCLE (Multiple Sequence Comparison by Log‐Expectation, (Edgar, 2004)) and phylogenetic trees were constructed using the Neighbour‐joining method with Jukes‐Cantor as the nucleotide distance measure and bootstrap analysis with 1,000 replicates. PRRSV‐2 VR2332 (PRU87392) was used as out‐group (CLC main Workbench v. 8.1.2 (QIAGEN).

The phylogenetic trees were visualized using FigTree v.1.4.3 (http://tree.bio.ed.ac.uk/software/figtree/).

Putative amino acid sequences were translated from corresponding nucleotide sequences using the translation‐to‐protein feature in CLC main workbench V. 8.1.2 (QIAGEN).

### 2.6 GenBank Accession numbers

Complete genome: DK‐2019‐10166‐107: Accession no. MN603982, ORF2‐7: DK‐2019‐10166‐100: Accession no. MN603983, DK‐2019‐10166‐106: Accession no. MN603984, DK‐2019‐10166‐109: Accession no. MN603985, DK‐2019‐11444‐17: Accession no. MN621889.

### 2.7 Recombination analyses

The recombination analysis was based on a MUSCLE alignment of 93 PRRSV‐1 sequences, including the ‘Horsens’ virus and the two MLV vaccine strains Amervac (Unistrain) and 96V198 (Suvaxyn), encoding ORF1‐ORF7.

For the recombination analysis, the Recombination Detection Program 4 v.4.97 (RDP4) (Martin, Murrell, Golden, Khoosal, & Muhire, 2015) was used. The seven algorithms utilized for the analysis were RDP, GENECONV, BootScan, MaxChi, Chimaera, SiScan, and 3Seq and were run with the default settings.

In addition, a sliding‐window analysis tool, SimPlot v3.5.1, was used to visualize the break point found from RDP4. The SimPlot was constructed with the settings: window: 500 base pairs, Step: 50 base pairs: GapStrip: On, Kimura (2‐parameter), T/t: 2.0.

### 2.8 Propagation of virus in cell culture

Viruses were isolated and propagated in Marc‐145 cells from one serum sample obtained from the boar station and one serum sample from a suckling pig from a multiplier farm. Serum was diluted 1:1 in Minimum Essential Media (MEM, Gibco) and filtrated through 0.45µm filter (Millex). Cells seeded in T25 flasks were inoculated with 0.5 ml filtrated serum and left in 37°C cell incubator, 5% CO_2_, for 2 hr to absorb. After incubation, the serum was removed and MEM supplemented with 5% FBS (Sigma), and 1% Pen/Strep/Glutamine (Gibco) were added to a final volume of 3 ml. The flasks were left for 5 days in 37°C cell incubator, 5% CO_2_. The virus isolates were harvest from one freeze/thaw cycle, and cell debris was removed by centrifugation at 500× ***g***, 10 min, RT. A second passages was obtained by inoculating T25 flasks with 200 µl 1st passage isolate and 3 ml MEM supplemented with 5% FBS (Sigma), 1% Pen/Strep/Glutamine (Gibco). The flasks were left in 37°C cell incubator, 5% CO_2_ for five days and harvest as described above. The virus isolates were stored at −80°C.

Tissue culture infectious dose 50 per ml (TCID50/ml) was determined by 10‐fold titration of the second passage on Marc‐145 cells seeded in 96‐well plates. Infected cells were visualized after fixation in absolute EtOH by staining using an immunoperoxidase monolayer assay (IPMA) as described previously (Bøtner, Nielsen, & Bille‐Hansen, 1994; Markussen & Have, 1992). PRRSV monoclonal antibody SDOW17‐A (RTI) was used as primary antibody. The TCID50/ml was calculated according to the method of Reed and Muench (Reed & Muench, 1938).

## 3 RESULTS

### 3.1 Test of samples from the boar station

On 22 July 2019, blood samples were obtained from 18 boars, distributed throughout all six sections, as a part of the routine surveillance program for PRRSV. The samples were tested for antibodies against PRRSV using a commercial ELISA (IDEXX PRRS X3 Ab Test). One of the samples tested positive for PRRSV and the boar station was immediately put under trade restrictions. On July 27, additional 24 blood samples were obtained from the section including the positive boar at of the first sampling. Of these 24 samples, 20 samples tested positive for antibodies against PRRSV‐1. Subsequent analyses of the samples by PRRSV specific real‐time RT‐PCR also generated positive results. Based on the results of retrospective test of samples taken for other purposes, it was predicted that the most likely infection time of the boar station was during the first week of July 2019. No clinical signs were noticed in the boar station.

### 3.2 Initial sequence analysis of samples from the boar station by cycle sequencing of ORF5

**Figure 1:**
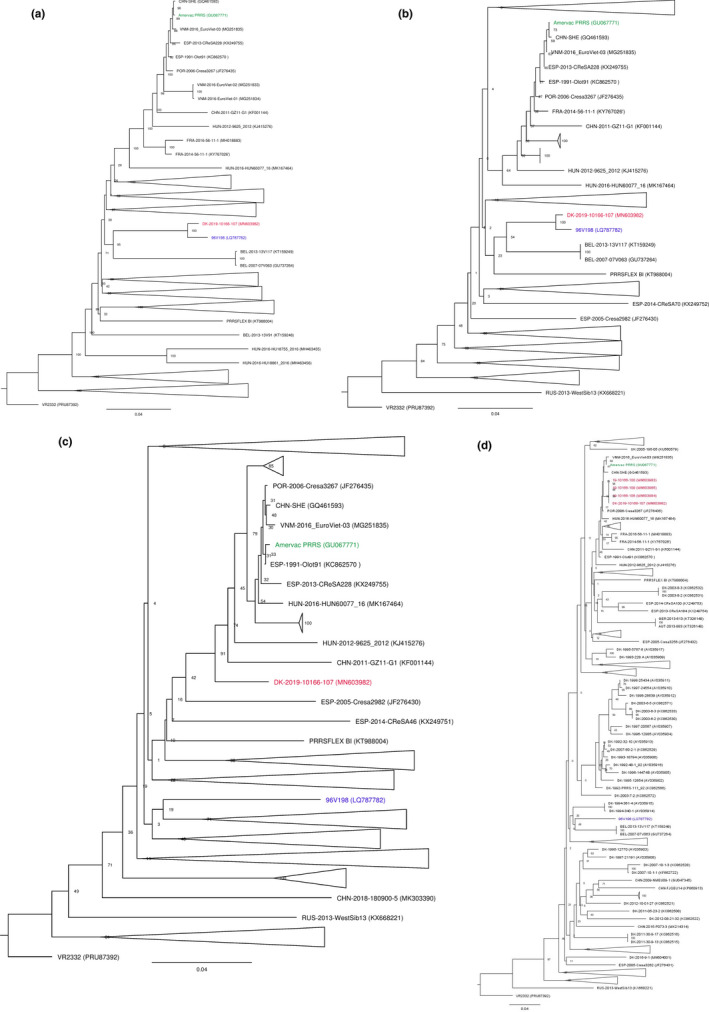
Phylogenetic analysis of the case virus with globally represented sequences. a. ORF1‐7, b. ORF2a, c. ORF3 and d. ORF5. The trees are shown as collapsed trees, for fully expanded trees see Figure S1a‐c and e. The phylogenetic trees were constructed using the Neighbour‐joining method with Jukes‐Cantor as the nucleotide distance measure and bootstrap analysis with 1,000 replicates. PRRSV‐2 VR2332 (PRU87392) was used as out‐group. Trees were drawn using FigTree v.1.4.3 [Colour figure can be viewed at wileyonlinelibrary.com]

For simplicity, the phylogenetic trees are shown as collapsed trees in the main text (Figure 1) and the full‐expanded trees are shown in Figures S1 and S2.

Initially, ORF5 sequences were obtained from four serum samples that tested positive for PRRSV‐1 by real‐time RT‐PCR. The phylogenetic analysis showed that the ORF5 sequences clustered together with the Amervac strain (the strain included in the Unistrain MLV vaccine from Hipra, Spain) (Figure 1d). Pairwise nucleotide comparisons revealed that the two strains were 99.01% identical. The closest matches to previously sequenced PRRSV‐1 from Denmark were to the strains DK‐2003‐8‐2 (KC862531) and DK‐2003‐8‐3 (KC862532), but the level of identity in ORF5 was only 90.76%. The four sequences from the boar station were 100% identical so only one of these isolates (DK‐2019‐10166‐107 or ‘Horsens virus’) was selected for further analyses.

Sequencing of ORF2‐ORF7 revealed that the DK‐2019‐10166‐107 (‘Horsens’) virus also showed high level of genetic similarity to the Amervac strain in ORF4‐ORF7 (99.01%–99.74%), but only 96.74% and 92.80% identity in ORF3 and ORF2, respectively (Table 1).

**Table 1:** Pairwise nucleotide and amino acid comparison of the case virus DK‐2019‐10166‐107 (MN603982) to the vaccine strains 96V198 and Amervac

ORF/protein	% identity to 96V198		% identity to Amervac	
	Nucleotides	Amino acids	Nucleotides	Amino acids
ORF1−7	98.06 (287)^a^	NA	91.95 (1,189)	NA
ORF1a/pp1a	99.62 (27)	99.25 (18)	91.41 (618)	91.44 (205)
ORF1b	99.73 (12)	NA	89.00 (483)	NA
ORF1ab/pp1ab	99.66 (39)	ND	90.48 (1,101)	ND
ORF2a/GP2	98.80 (9)	97.19 (7)	92.80 (54)	93.98 (15)
ORF2b/E	95.77 (9)	97.14 (2)	98.59 (3)	95.71 (3)
ORF3/GP3	90.10 (79)	90.57 (25)	96.74 (26)	94.72 (14)
ORF4/GP4	87.14 (71)	85.79 (26)	99.09 (5)	98.91 (2)
ORF5/GP5	88.84 (67)	91.04 (18)	99.01 (6)	97.51 (5)
ORF5a/ORF5a protein	90.91 (12)	86.05 (6)	99.24 (1)	100 (0)
ORF6/M	92.91 (37)	94.22 (10)	99.43 (3)	98.84 (2)
ORF7/N	93.28 (26)	92.97 (9)	99.74 (1)	99.22 (1)

Abbreviations: NA, Not applicable; ND, Not done.

^a^ Number of amino acid and nucleotides differences are shown in brackets.

### 3.3 Full genome sequencing of the ‘Horsens’ virus

Full genome sequencing by NGS using the Miseq (Illumina) platform was performed on the same serum sample as the one selected above—the ‘Horsens strain’ DK‐2019‐10166‐107 (MN603982). The quality of the sequencing data was assessed by the FastQC applicant (v0.11.8), and the trimming of reads was done accordingly using CLC Genomics v11.0.1. The trimmed data were assembled to the Amervac vaccine strain (GU067771) resulting in a sequence of 15,098 nucleotides including the 5′‐ and 3′‐UTRs. A Blastn search for ORF1‐ORF7 found the best match to Lelystad virus (M96262) with 92.66% identity. The sequence obtained from NGS was 100% identical in ORF2‐7 to the same sample sequenced by cycle sequencing.

The results of the pairwise nucleotide and amino acid comparisons of the separate ORFs and proteins are shown in Table 1. The phylogenetic analyses were repeated on ORFs1‐7 combined and separately on the single ORFs. The phylogenetic analysis of the combined ORFs1‐7 revealed that the ‘Horsens’ virus grouped with the 96V198 strain, which is the strain included in the Suvaxyn PRRS MLV vaccine (Figure 1a; S1 and S2). Interestingly, the ‘Horsens’ virus grouped with the Amervac strain when the analyses were performed on ORF4 to ORF7 separately (Figure 1d; S1 and S2), whereas it grouped with the 96V198 strain in ORF2a (Figure 1b). In ORF3, the Horsens virus grouped between the two MLV vaccine strains (Figure 1c; S1 and S2).

These results indicated that the virus was a recombination between different viruses and therefore a recombination analyses was carried out.

### 3.4 Recombination analyses of the Horsens strain

The recombination analysis of the ‘Horsens’ strain DK‐2019‐10166‐107 (MN603982) was conducted on a multi‐sequence alignment (93 sequences) of ORF1‐7 using RDP4. All seven algorithms utilized for the break point detection agreed with high probability that DK‐2019‐10166‐107 was a recombinant virus (Table 2). The program predicted a break point at nucleotide position 12,383 (measured from first nucleotide in ORF1a) with the 96V198 strain as the major parent and the Amervac strain as the minor parent. No ending break point was identified. Pairwise nucleotide comparison of the recombinant virus with the two parental viruses downstream and upstream of the putative recombination break point showed a high level of similarity (Table 3).

**Table 2:**
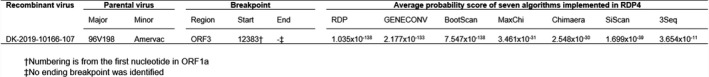
Overview of putative recombination breakpoint and probability of predicted breakpoint from seven algorithms implemented in RDP4

**Table 3:** Pairwise nucleotide comparison downstream and upstream of putative recombination break point

Nucleotide position	Identity % to 96V198	Identity % to Amervac
1–12383	99.60 (49)^a^	90.55 (1,170)
12384–14763	90.00 (238)	99.20 (19)

^a^ Number of nucleotide differences are shown in brackets. Numbering is from the first nucleotide in ORF1a.

**Figure 2:**
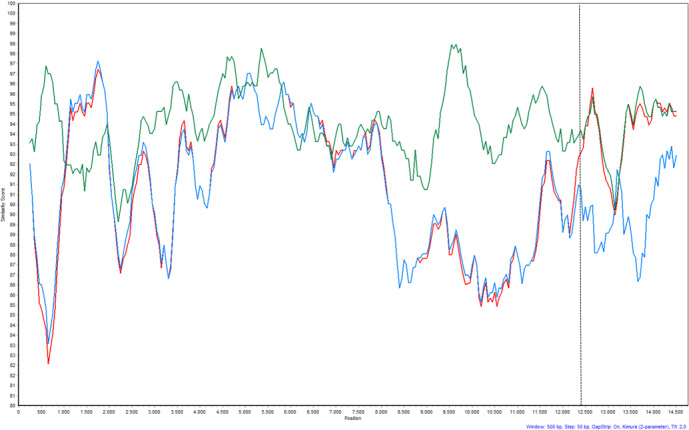
SimPlot analysis of the major parent virus, the minor parent virus and the recombinant virus. Blue line: 96V198, Green line: Amervac and Red line: DK‐2019‐10166‐107. Putative starting break point at nucleotide position 12,383 for recombination is marked with dashed lines. Y‐axis: Similarity score, x‐axis: nucleotide position counted from 1. nucleotide in ORF1a [Colour figure can be viewed at wileyonlinelibrary.com]

A Simplot analysis of the major parent, minor parent and recombinant virus further supported the break point (Figure 2).

**Figure 3:**
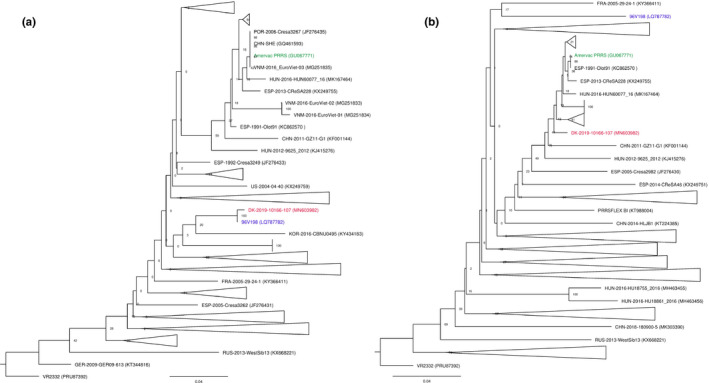
Phylogenetic trees of ORF3. a. downstream (nucleotide 1–201) and b. upstream (nucleotide 202–798) of putative break point. The trees are shown as collapsed trees, for fully expanded trees see Figure S2. The phylogenetic trees were constructed using the Neighbor Joining method with Jukes‐Cantor as the nucleotide distance measure and bootstrap analysis with 1,000 replicates. PRRSV‐2 VR2332 (PRU87392) was used as outgroup. Trees were drawn using FigTree v.1.4.3 [Colour figure can be viewed at wileyonlinelibrary.com]

The break point is located in ORF3 encoding the glycoprotein 3 (GP3) after position nucleotide 201 corresponding to amino acid 67. Pairwise nucleotide and amino acid comparison of the recombinant virus and the two parental viruses further confirmed the predicted break point (Table 4). A phylogenetic analysis of ORF3 downstream (nucleotide 1–201) and upstream (nucleotide 202–798) of the break point further sustains that the ‘Horsens virus’ DK‐2019‐10166‐107 is a recombination between the 96V198 strain and the Amervac strain (Figure 3).

**Table 4:** Pairwise amino acid and nucleotide comparison downstream and upstream of putative recombination break point in GP3/ORF3

Amino acid position	Identity % to 96V198	Identity % to Amervac
1–67	100 (0)^a^	85.07 (10)
68–265	87.37 (25)	97.98 (4)

Nucleotide position	Identity % to 96V198	Identity % to Amervac
1–201	99.50 (1)	90.05 (20)
202–798	86.93 (78)	98.99 (6)

^a^ Number of amino acid and nucleotides differences are shown in brackets.

### 3.5 Isolation of virus from cell culture and sequencing

To confirm the stability of the recombinant virus in vitro*,* one serum sample obtained from the boar station (DK‐2019‐10166‐107 passage 2) and one serum sample obtained from a suckling pig originating from a production farm, where sows were infected by semen (DK‐2019‐11444‐17 passage 2) were propagated in two passages in Marc‐145 cells. Titres of the 2nd passage isolates were calculated to 1.58 × 10^6^ and 5.0 × 10^4^ TCID50/ml for the boar and piglet, respectively. ORF2‐7 sequences from both isolates were obtained by cycle sequencing and compared to the index strain DK‐2019‐10166‐107 (MN603982) sequenced by NGS directly from serum. The sequences obtained from the boar were still 100% identical, and the sequence obtained from the piglet was still 99.97% identical to the boar sequence (1 nucleotide difference) confirming the stability of the recombinant virus also after propagation in cell culture. Isolation in pulmonary aleveolar marcrophages (PAM) was not attempted since the virus grew well in MARC cells.

### 3.6 Test of samples from the neighbouring farm

In total, 21 of 50 serum samples, that were collected from a neighbouring herd, which had used both vaccines within the last 12 months, tested positive for PRRSV‐1 by real‐time RT‐PCR. Sequencing of ORF2 and ORF5 from 14 of these samples revealed that both the 96V198 and the Amervac virus strains were circulating in the herd. In addition, one sample was 99.81% identical to the Horsens virus DK‐2019‐10166‐107 in ORFs2‐7 and by that, also indicating circulation of the recombinant strain.

### 3.7 Results from herds receiving semen from the PRRSV infected boar station

Three of the 71 PRRSV free nucleus and multiplier herds that received semen during July 2019 were found to be PRRSV infected based on serological analyses, real‐time RT‐PCR tests and sequencing. Until now (primo November 2019), 38 of the production herds that had received semen during July 2019 have been infected with the recombinant strain based on cycle sequencing of partial ORF2 (488–684 nucleotides) and full ORF5. The analyses of these sequences revealed minimum 99.7% nucleotide identity to the DK‐2019‐10166‐107 index strain.

Statistical evaluation of the production data including farrowing rate, number of live‐born piglets and other clinical signs from the herds is pending. Based on personal communications from the herd veterinarians, fever and anorexia were evident in sows in addition to a decreased milk production and increased piglet mortality up to 60%. Furthermore, one of the infected herds submitted processing fluid from two‐day‐old sick piglets and another submitted lung samples from ten piglets that died shortly after birth. All of these samples were strongly positive for PRRSV when tested by real‐time RT‐PCR—some with Ct values below 12, which is much lower than usual seen in acutely PRRSV‐1 infected animals.

## 4 DISCUSSION

This paper describes an outbreak of PRRSV‐1 in Denmark that started in a PRRSV‐negative boar station. The boar station is situated in a relatively swine dense area, and therefore, it is not surprising that outbreaks can occur, despite that the level of external biosecurity was high. This is nevertheless the first PRRSV outbreak for more than 17 years in any boar station in Denmark.

The results of the genetic characterization of the virus from the boar station underlined that sequencing of a small part of the PRRSV genome may lead to false conclusions and that the results of sequence analyses using programs such as BLAST may be misleading: initially, the ORF5 of the ‘Horsens’ virus was sequenced and revealed >99% identity to the Amervac strain that are included in the Unistrain PRRSV vaccine from Hipra, Spain. This indicated that the boar station was infected by the vaccine strain and implied that further transmission by semen would be limited since this strain is attenuated. However, subsequent full genome characterization of the strain revealed that the virus was indeed almost identical to the Amervac strain in ORFs 4–7, but the sequences of ORFs1‐2 and part of ORF3 differed significantly from the Amervac strain (approx. 7% differences at the nucleotide level). ‘Blastn’ analyses of the ORF2 sequence revealed that the highest level of identity of the ‘Horsens’ strain was to a Belgian strain (94V360), but still, this strain differed more than 6% from the ‘Horsens’ strain. Since the name of the ‘best match’ Belgian strain resembled the strain included in the Suvaxyn vaccine (96V198), we performed a search in ‘GenBank – nucleotide search’ with the search word ‘96V198’. This search generated several hits on sequences of the 96V198 strain. These sequences were annotated as ‘patented strains’ and were not picked up by the Blastn search. This surprisingly revealed that the ‘Blastn’ program does not pick up all sequences in GenBank database despite that the sequences were deposited and were public accessible.

Pairwise comparison of the 96V198 strain and the ‘Horsens’ strain revealed that the two strains were almost identical in ORFs 1 and 2. Subsequently, we contacted Zoetis AH, who generously provided the full genome sequence of the 96V198 strain and repeated the analyses. As described in the paper, the results of these analyses revealed that the ‘Horsens’ strain is a recombination between the Amervac vaccine strain, included in the Unistrain PRRS vaccine, and the 96V198 strain included in the Suvaxyn PRRS vaccine. The major parent is the 96V198 strain that encompasses ORFs 1–2 and part of ORF 3 (12,383 nucleotides) whereas the Amervac part constitute the minor parent (2,380 nucleotides) spanning ORFs 3–7. The high level of genetic identity of the recombinant virus and the parent vaccine strains indicate that the recombination event has happened recently.

Recombination between PRRSV field strains has been extensively described for PRRSV‐2 (Dong et al., 2017; Li et al., 2009; Wenhui et al., 2012; Zhao et al., 2017) and less frequently for PRRSV‐1 (Dortmans, Buter, Dijkman, Houben, & Duinhof, 2019; Martin‐Valls et al., 2014). More rarely, recombination between PRRSV‐1 field strains and strains of PRRSV‐1 MLV vaccine strains has been reported (Chen et al., 2017; Frossard et al., 2013; Marton et al., 2019). In two of these reports, the Unistrain vaccine strain Amervac was part of the recombined virus, but the recombination break point differed from the ‘Horsens’ virus. Recombination between two PRRSV‐1 vaccine strains has only been reported once from France where a recombinant between the ‘DV’ vaccine strain (Porcilis PRRS, MSD) and the ‘Amervac’ strain (Unistrain, Hipra) was detected in a single herd (Renson et al., 2017).

For some of the PRRSV‐2 recombinant viruses, there seems to be an increased pathogenicity of the recombinant strains compared to their parental strains (Bian et al., 2017; Lu et al., 2015). In contrast, a recent study did not find a correlation between the recombinant phenotypes and pathogenicity (van Geelen et al., 2018). An experimental study from France revealed that the Porcilis/Unistrain recombinant virus describe above (Renson et al., 2017) seems to be more virulent than the two parent PRRSV‐1 vaccine viruses (Eclercy et al., 2019). However, this study did not assess if the increased virulence of the recombinant virus reflected that the recombinant virus was adapted to swine or if it was a result of the recombination per se*.* In general, there are limiting data that link specific genetic traits with pathogenicity for any PRRSV strain (van Geelen et al., 2018). Nevertheless, data from experimental infections sustain that some PRRSV‐1 strains have increased virulence, measured as aggravating clinical signs, severe macroscopic lung lesions and/or enhanced onset, peak and duration of viremia (Karniychuk et al., 2010; Sinn et al., 2016; Stadejek et al., 2017). During the outbreak in the boar station, there were no clinical signs of disease, which is normal when adult, non‐pregnant animals are exposed to PRRSV (Nathues et al., 2014; Prieto & Castro, 2005). In contrast, reports from the field indicate that ‘Horsens’ virus is causing more severe disease than normally observed in Danish herds infected with PRRSV‐1 strains. The clinical signs include severe reproductive problems and abortions, significant increase in post farrowing mortality and respiratory disease in young pigs. Furthermore, the load of virus found in processing fluids, lungs and serum of infected pigs exceed the levels normally seen in samples from PRRSV diseased pigs, which indicate a very high level of viral replication. Compiled, the epidemiological, virological and clinical data indicate that this strain has regained a profound level of virulence despite that the two close‐related parent viruses are attenuated vaccine strains. Thus, these findings suggested that recombination may generate new strains with higher virulence than the parent strains. As discussed above, there is a lack of data linking genetic data with difference in virulence, but it is striking that this recombinant strain shares such a high level of identity to the attenuated parent strains and still seems to be causing sustained disease in the field. There are small genomic areas where the recombinant strain is different from both parent strains, which may account for the apparent reversion to virulence, but, again, this is purely speculative. These differences are probably due to mutations acquired by the recombinant strain during pig passage; however, the possibility that a more virulent field strain also participated in the recombination event cannot be excluded although this was not picked up by any of the seven algorithms utilized for the recombination analysis. The only protein that is significantly different from all other known PRRSV‐1 viruses is the GP3 protein which is a hybrid between the two parent strains. Still, the first 67 amino acids of the recombinant GP3 are 100% identical to the 96V198 strain, and in the remaining part of the protein (residues 68–265), there are only four amino acid differences to the Amervac strain—all situated in a previously defined hypervariable site of GP3 (Oleksiewicz, Bøtner, & Normann, 2002). In conclusion, we have not been able to identify obvious genetic traits that can explain the apparent change in virulence of this strain. Furthermore, we have previously experienced severe outbreaks with PRRSV‐2 strains that could not be reproduced experimentally (Kvisgaard et al., 2017), emphasizing that proper evaluation of the virulence of PRRSV strains requires controlled experimental trials.

Sequencing of samples from a neighbouring herd revealed that both vaccine strains and the recombinant strain were simultaneously circulating at this farm. These results, combined with the information that one of the involved vaccines has been used in only two other herds in Denmark, and the fact that the recombinant virus is closely related to the vaccine strains, strongly indicate that the recombinant virus evolved in this herd and the virus subsequently spread to the boar station. However, since the samples were taken at the neighbouring herd after the virus was detected at the boar station, the directionality of transmission cannot be determined.

The source of introduction to the boar station is not clear, but the best guess is that the virus was introduced by air since the boar station has not established filtration on incoming airflow. The neighbouring herd is situated 5.8 km west of the boar station. Wind borne transmission of PRRSV‐2 strains for up to 9 km has been described in the United States (Dee, Otake, Oliveira, & Deen, 2009; Otake, Dee, Corzo, Oliveira, & Deen, 2010). The test of samples taken retrospectively at the boar station indicated that the herd was infected between 1 July 2019 and 10 July 2019. Interestingly, according to data from the Danish metrological institute (www.dmi.dk), the wind directions during this time period were ‘west’ or ‘north‐west’, the average temperature was 12–14 degree Celsius and the relative humidity was 78%–80%. Half‐lives of aerosolized infectious PRRSV at these environmental conditions have previously been predicted to be between 50 and 80 min which make airborne transmission plausible (Hermann et al., 2007). Since it took up to 3 weeks before the distribution of semen was terminated, more than 700 herds received semen that may have been contaminated with PRRSV. Indeed, sequencing of partial ORF2 and full ORF5 from 38 of these herds confirmed that these herds subsequently became infected with a virus identical to the ‘Horsens’ virus, but the exact number of infected herds is not known since some herds may have submitted samples to other laboratories.

Transmission of PRRSV‐1 by semen is well described both under experimental conditions (Prieto & Castro, 2005) and in the field (Nathues et al., 2014), and this outbreak emphasizes that a very tight surveillance program is needed to mitigate the impact of PRRSV outbreaks at boar stations. The boar station was tested by bi‐monthly blood samples; however, the samples were only tested for antibodies against PRRSV. Antibody responses to PRRSV are measurable 7–10 days after infection, and therefore, the infection in this boar station remained undetected for almost three weeks. Indeed, one of the serum samples taken two weeks earlier was retrospectively found to be positive for PRRSV by PCR, but was negative for antibodies. This empathizes that optimized surveillance programs should include test for both antibodies and virus; tests should be performed as frequent as possible—and at least on a weekly basis.

## 5 CONCLUSION

We have identified a PRRSV‐1 virus that has evolved from a homologous recombination event between two PRRSV‐1 MLV vaccine strains. The virus seems to be highly transmissible and causes severe disease in infected herds despite that the virus shares high level of genetic identity to the attenuated parent vaccine strains. The source of infection is presumable a neighbouring farm situated 5.8 km from the boar station. Further studies are in progress to assess the virulence of the virus in controlled experimental trials.

## CONFLICT OF INTERESTS

Two of the eight authors work for SEGES Danish Pig Research Centre. The aim of the Danish Pig Research Centre is to safeguard the interests of the Danish pig producers.

## Ethical approval

The authors confirm that the ethical policies of the journal, as noted on the journal's author guidelines page, have been adhered to. No ethical approval was required as this report solely contains diagnostic samples taken in the field.

## Supporting information

Supplementary Material

## Data Availability

The data that support the findings of this study are available from the corresponding author upon reasonable request.
