# Supplementary material for: A recombination between two Type 1 Porcine Reproductive and Respiratory Syndrome Virus (PRRSV‐1) vaccine strains has caused severe outbreaks in Danish pigs
Source: Transbound Emerg Dis. 2020 May 25;67(5):1786–96. doi: 10.1111/tbed.13555 (PMC7540543; doi:10.1111/tbed.13555)
Supplement: Supplementary file 1 — Supplementary Material [file TBED-67-1786-s001.pdf]

Supplementary Table 1

Supplementary Table 1. Primers and conventional PCR conditions

Primer	Sequence 5'-3'	Annealing temp.	Elongation time (min)	Size bp
**Primers for individually ORF2 to ORF7 amplification:**				
ORF2	**Fw:** CTGGCACAGAATTGCAGGTA<br>**Rev:** GCACACTGATGAGCCATTGT	55	1	697
ORF2.3	**Fw:** TGCTCCGCGCTTCTCCGTTCG<br>**Rev:** ACATAGCGTAGAGCTGGAATTCG	55	1	552
ORF3	**Fw:** ACAATGGCTCATCAGTGTGC<br>**Rev:** TGAAGCCTTTCTCGCTCATT	55	1	900
ORF4	**Fw:** AGCGTGACCATGATGAGTTG<br>**Rev:** AAAAGCCACCAGAAGCAAGA	55	1	902
ORF5	**Fw:** TGAGGTGGGCTACAACCATT<br>**Rev:** AGGCTAGCACGAGCTTTTGT	55	1	703
ORF6	**Fw:** GTCCTCGAAGGGGTTAAAGC<br>**Rev:** CTGTCCTCCCCTAGGTTGCT	55	1	695
ORF7	**Fw:** GGCAAACGAGCTGTTAAACG<br>**Rev:** AATTTCGGTCACATGGTTCC	55	1	544
**Long range PCR for full genome sequencing:**				
A	**Fw:** GGCGCGCCTAATACGACTCACT-ATAGATGATGTGTAGGGTATTCCCC-CTACATACACGACA<br>**Rev:** CGC GGG CGC TTG AGT TCG GCA AAT T	50	4	3019
B	**Fw:** CCTGGACCAGCCTTTAAATC<br>**Rev:** TCCGTGTAAAAGGTGTCACC	50	6	4332
C	**Fw:** CCC CTC TTT TTG AGA ATG GT<br>**Rev:** ACATAGCGTAGAGCTGGAATTCG	50	6	5602
D	**Fw:** CTGGCACAGAATTGCAGGTA<br>**Rev:** AATTTCGGTCACATGGTTCC	50	4	2695

Supplementary Figure 1

Supplementary Figure 1: Phylogenetic analysis of the case virus with globally represented sequences. a. ORF1-7, b. ORF2a, c. ORF3, d. ORF4, e. ORF5, f. ORF6, g. ORF7. The phylogenetic trees were constructed using the Neighbor Joining method with Jukes-Cantor as the nucleotide distance measure and bootstrap analysis with 1000 replicates. PRRSV-2 VR2332 (PRU87392) was used as outgroup. Trees were drawn using FigTree v.1.4.3.

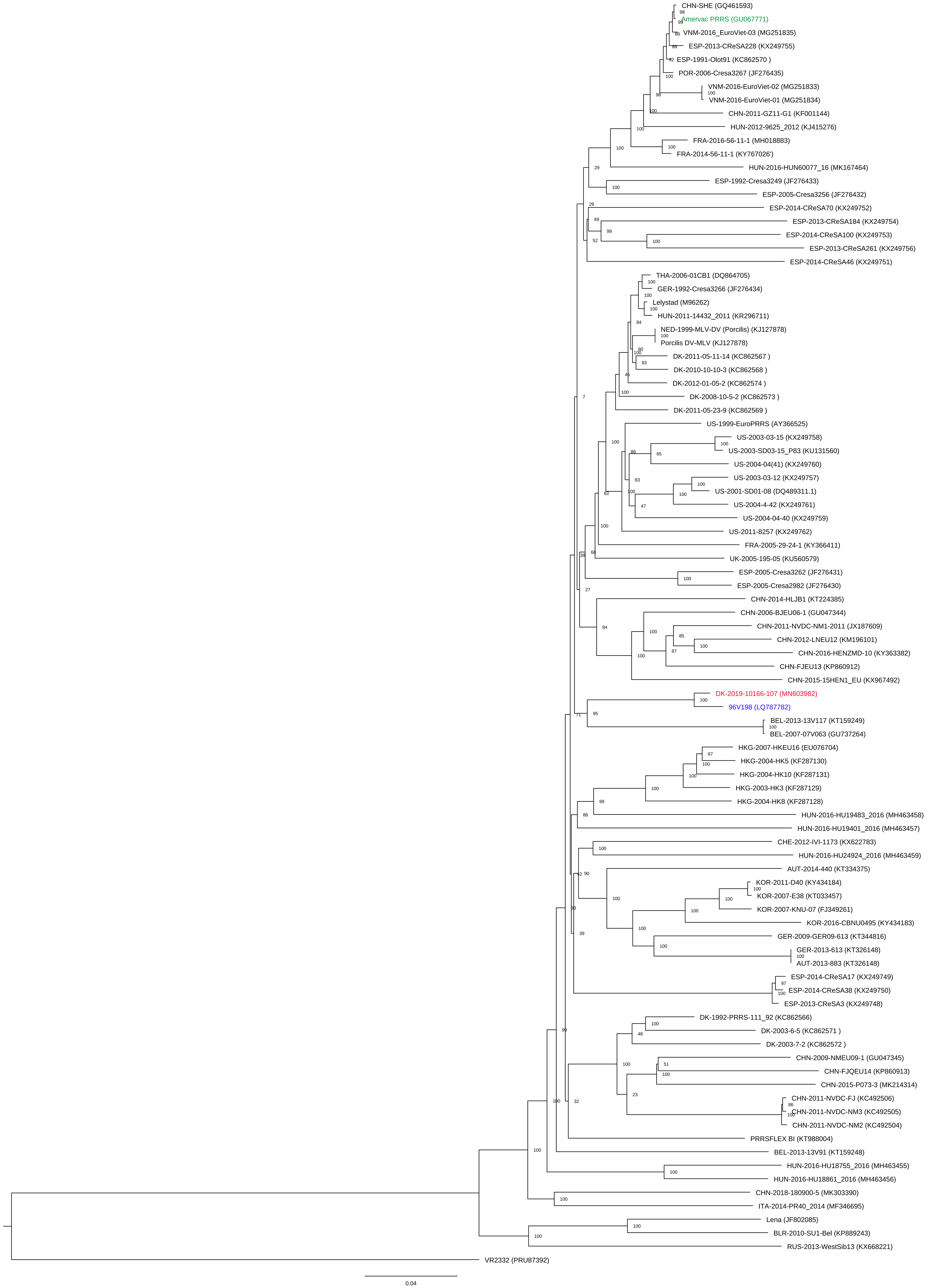

CHN-SHE (GQ461593)
Amervac PRRS (GU067771)
VNM-2016_EuroViet-03 (MG251835)
ESP-2013-CReSA228 (KX249755)
ESP-1991-Olot91 (KC862570)
POR-2006-Cresa3267 (JF276435)
VNM-2016-EuroViet-02 (MG251833)
VNM-2016-EuroViet-01 (MG251834)
CHN-2011-GZ11-G1 (KF001144)
HUN-2012-9625_2012 (KJ415276)
FRA-2016-56-11-1 (MH018883)
FRA-2014-56-11-1 (KY767026')
HUN-2016-HUN60077_16 (MK167464)
ESP-1992-Cresa3249 (JF276433)
ESP-2005-Cresa3256 (JF276432)
ESP-2014-CReSA70 (KX249752)
ESP-2013-CReSA184 (KX249754)
ESP-2014-CReSA100 (KX249753)
ESP-2013-CReSA261 (KX249756)
ESP-2014-CReSA46 (KX249751)
THA-2006-01CB1 (DQ864705)
GER-1992-Cresa3266 (JF276434)
Lelystad (M96262)
HUN-2011-14432_2011 (KR296711)
NED-1999-MLV-DV (Porcilis) (KJ127878)
Porcilis DV-MLV (KJ127878)
DK-2011-05-11-14 (KC862567)
DK-2010-10-10-3 (KC862568)
DK-2012-01-05-2 (KC862574)
DK-2008-10-5-2 (KC862573)
DK-2011-05-23-9 (KC862569)
US-1999-EuroPRRS (AY366525)
US-2003-03-15 (KX249758)
US-2003-SD03-15_P83 (KU131560)
US-2004-04(41) (KX249760)
US-2003-03-12 (KX249757)
US-2001-SD01-08 (DQ489311.1)
US-2004-4-42 (KX249761)
US-2004-04-40 (KX249759)
US-2011-8257 (KX249762)
FRA-2005-29-24-1 (KY366411)
UK-2005-195-05 (KU560579)
ESP-2005-Cresa3262 (JF276431)
ESP-2005-Cresa2982 (JF276430)
CHN-2014-HLJB1 (KT224385)
CHN-2006-BJEU06-1 (GU047344)
CHN-2011-NVDC-NM1-2011 (JX187609)
CHN-2012-LNEU12 (KM196101)
CHN-2016-HENZMD-10 (KY363382)
CHN-FJEU13 (KP860912)
CHN-2015-15HEN1_EU (KX967492)
DK-2019-10166-107 (MN603982)
96V198 (LQ787782)
BEL-2013-13V117 (KT159249)
BEL-2007-07V063 (GU737264)
HKG-2007-HKEU16 (EU076704)
HKG-2004-HK5 (KF287130)
HKG-2004-HK10 (KF287131)
HKG-2003-HK3 (KF287129)
HKG-2004-HK8 (KF287128)
HUN-2016-HU19483_2016 (MH463458)
HUN-2016-HU19401_2016 (MH463457)
CHE-2012-IVI-1173 (KX622783)
HUN-2016-HU24924_2016 (MH463459)
AUT-2014-440 (KT334375)
KOR-2011-D40 (KY434184)
KOR-2007-E38 (KT033457)
KOR-2007-KNU-07 (FJ349261)
KOR-2016-CBNU0495 (KY434183)
GER-2009-GER09-613 (KT344816)
GER-2013-613 (KT326148)
AUT-2013-883 (KT326148)
ESP-2014-CReSA17 (KX249749)
ESP-2014-CReSA38 (KX249750)
ESP-2013-CReSA3 (KX249748)
DK-1992-PRRS-111_92 (KC862566)
DK-2003-6-5 (KC862571)
DK-2003-7-2 (KC862572)
CHN-2009-NMEU09-1 (GU047345)
CHN-FJQEU14 (KP860913)
CHN-2015-P073-3 (MK214314)
CHN-2011-NVDC-FJ (KC492506)
CHN-2011-NVDC-NM3 (KC492505)
CHN-2011-NVDC-NM2 (KC492504)
PRRSFLEX BI (KT988004)
BEL-2013-13V91 (KT159248)
HUN-2016-HU18755_2016 (MH463455)
HUN-2016-HU18861_2016 (MH463456)
CHN-2018-180900-5 (MK303390)
ITA-2014-PR40_2014 (MF346695)
Lena (JF802085)
BLR-2010-SU1-Bel (KP889243)
RUS-2013-WestSib13 (KX668221)
VR2332 (PRU87392)
0.04

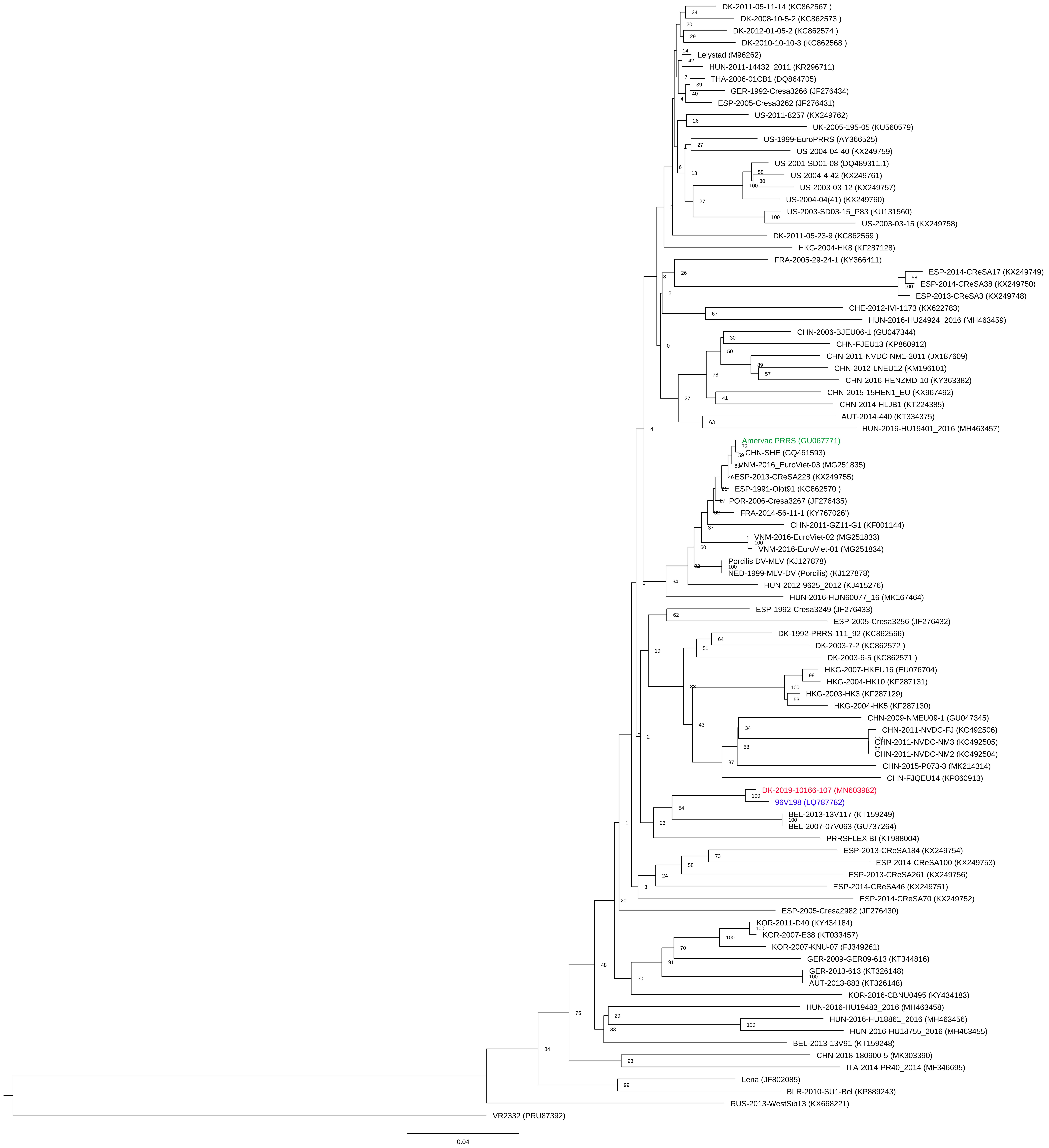
DK-2011-05-11-14 (KC862567)
DK-2008-10-5-2 (KC862573)
DK-2012-01-05-2 (KC862574)
DK-2010-10-10-3 (KC862568)
Lelystad (M96262)
HUN-2011-14432_2011 (KR296711)
THA-2006-01CB1 (DQ864705)
GER-1992-Cresa3266 (JF276434)
ESP-2005-Cresa3262 (JF276431)
US-2011-8257 (KX249762)
UK-2005-195-05 (KU560579)
US-1999-EuroPRRS (AY366525)
US-2004-04-40 (KX249759)
US-2001-SD01-08 (DQ489311.1)
US-2004-4-42 (KX249761)
US-2003-03-12 (KX249757)
US-2004-04(41) (KX249760)
US-2003-SD03-15_P83 (KU131560)
US-2003-03-15 (KX249758)
DK-2011-05-23-9 (KC862569)
HKG-2004-HK8 (KF287128)
FRA-2005-29-24-1 (KY366411)
ESP-2014-CReSA17 (KX249749)
ESP-2014-CReSA38 (KX249750)
ESP-2013-CReSA3 (KX249748)
CHE-2012-IVI-1173 (KX622783)
HUN-2016-HU24924_2016 (MH463459)
CHN-2006-BJEU06-1 (GU047344)
CHN-FJEU13 (KP860912)
CHN-2011-NVDC-NM1-2011 (JX187609)
CHN-2012-LNEU12 (KM196101)
CHN-2016-HENZMD-10 (KY363382)
CHN-2015-15HEN1_EU (KX967492)
CHN-2014-HLJB1 (KT224385)
AUT-2014-440 (KT334375)
HUN-2016-HU19401_2016 (MH463457)
Amervac PRRS (GU067771)
CHN-SHE (GQ461593)
VNM-2016_EuroViet-03 (MG251835)
ESP-2013-CReSA228 (KX249755)
ESP-1991-Olot91 (KC862570)
POR-2006-Cresa3267 (JF276435)
FRA-2014-56-11-1 (KY767026')
CHN-2011-GZ11-G1 (KF001144)
VNM-2016-EuroViet-02 (MG251833)
VNM-2016-EuroViet-01 (MG251834)
Porcilis DV-MLV (KJ127878)
NED-1999-MLV-DV (Porcilis) (KJ127878)
HUN-2012-9625_2012 (KJ415276)
HUN-2016-HUN60077_16 (MK167464)
ESP-1992-Cresa3249 (JF276433)
ESP-2005-Cresa3256 (JF276432)
DK-1992-PRRS-111_92 (KC862566)
DK-2003-7-2 (KC862572)
DK-2003-6-5 (KC862571)
HKG-2007-HKEU16 (EU076704)
HKG-2004-HK10 (KF287131)
HKG-2003-HK3 (KF287129)
HKG-2004-HK5 (KF287130)
CHN-2009-NMEU09-1 (GU047345)
CHN-2011-NVDC-FJ (KC492506)
CHN-2011-NVDC-NM3 (KC492505)
CHN-2011-NVDC-NM2 (KC492504)
CHN-2015-P073-3 (MK214314)
CHN-FJQEU14 (KP860913)
DK-2019-10166-107 (MN603982)
96V198 (LQ787782)
BEL-2013-13V117 (KT159249)
BEL-2007-07V063 (GU737264)
PRRSFLEX BI (KT988004)
ESP-2013-CReSA184 (KX249754)
ESP-2014-CReSA100 (KX249753)
ESP-2013-CReSA261 (KX249756)
ESP-2014-CReSA46 (KX249751)
ESP-2014-CReSA70 (KX249752)
ESP-2005-Cresa2982 (JF276430)
KOR-2011-D40 (KY434184)
KOR-2007-E38 (KT033457)
KOR-2007-KNU-07 (FJ349261)
GER-2009-GER09-613 (KT344816)
GER-2013-613 (KT326148)
AUT-2013-883 (KT326148)
KOR-2016-CBNU0495 (KY434183)
HUN-2016-HU19483_2016 (MH463458)
HUN-2016-HU18861_2016 (MH463456)
HUN-2016-HU18755_2016 (MH463455)
BEL-2013-13V91 (KT159248)
CHN-2018-180900-5 (MK303390)
ITA-2014-PR40_2014 (MF346695)
Lena (JF802085)
BLR-2010-SU1-Bel (KP889243)
RUS-2013-WestSib13 (KX668221)
VR2332 (PRU87392)
0.04

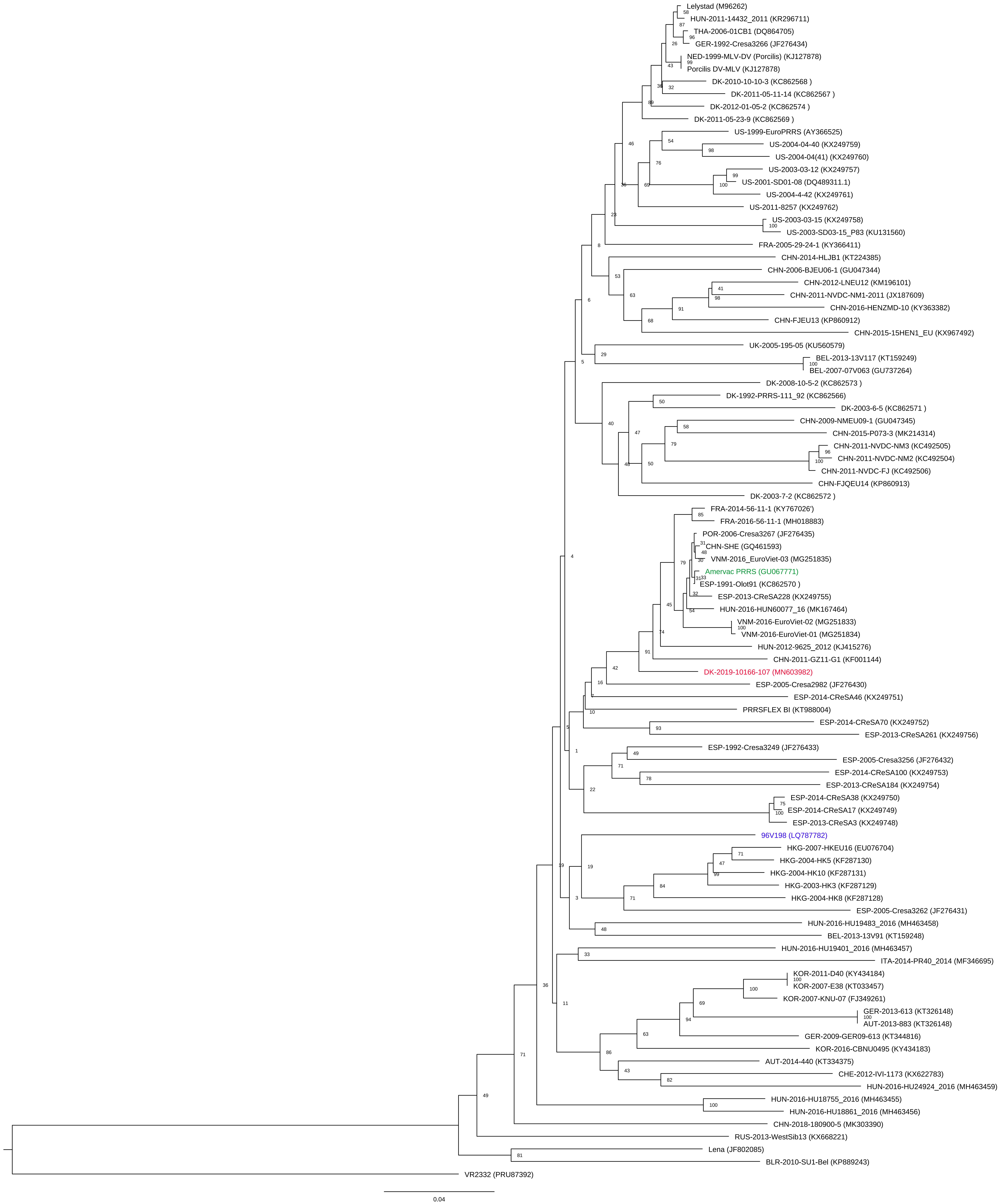

Lelystad (M96262)
HUN-2011-14432_2011 (KR296711)
THA-2006-01CB1 (DQ864705)
GER-1992-Cresa3266 (JF276434)
NED-1999-MLV-DV (Porcilis) (KJ127878)
Porcilis DV-MLV (KJ127878)
DK-2010-10-10-3 (KC862568)
DK-2011-05-11-14 (KC862567)
DK-2012-01-05-2 (KC862574)
DK-2011-05-23-9 (KC862569)
US-1999-EuroPRRS (AY366525)
US-2004-04-40 (KX249759)
US-2004-04(41) (KX249760)
US-2003-03-12 (KX249757)
US-2001-SD01-08 (DQ489311.1)
US-2004-4-42 (KX249761)
US-2011-8257 (KX249762)
US-2003-03-15 (KX249758)
US-2003-SD03-15_P83 (KU131560)
FRA-2005-29-24-1 (KY366411)
CHN-2014-HLJB1 (KT224385)
CHN-2006-BJEU06-1 (GU047344)
CHN-2012-LNEU12 (KM196101)
CHN-2011-NVDC-NM1-2011 (JX187609)
CHN-2016-HENZMD-10 (KY363382)
CHN-FJEU13 (KP860912)
CHN-2015-15HEN1_EU (KX967492)
UK-2005-195-05 (KU560579)
BEL-2013-13V117 (KT159249)
BEL-2007-07V063 (GU737264)
DK-2008-10-5-2 (KC862573)
DK-1992-PRRS-111_92 (KC862566)
DK-2003-6-5 (KC862571)
CHN-2009-NMEU09-1 (GU047345)
CHN-2015-P073-3 (MK214314)
CHN-2011-NVDC-NM3 (KC492505)
CHN-2011-NVDC-NM2 (KC492504)
CHN-2011-NVDC-FJ (KC492506)
CHN-FJQEU14 (KP860913)
DK-2003-7-2 (KC862572)
FRA-2014-56-11-1 (KY767026')
FRA-2016-56-11-1 (MH018883)
POR-2006-Cresa3267 (JF276435)
CHN-SHE (GQ461593)
VNM-2016_EuroViet-03 (MG251835)
Amervac PRRS (GU067771)
ESP-1991-Olot91 (KC862570)
ESP-2013-CReSA228 (KX249755)
HUN-2016-HUN60077_16 (MK167464)
VNM-2016-EuroViet-02 (MG251833)
VNM-2016-EuroViet-01 (MG251834)
HUN-2012-9625_2012 (KJ415276)
CHN-2011-GZ11-G1 (KF001144)
DK-2019-10166-107 (MN603982)
ESP-2005-Cresa2982 (JF276430)
ESP-2014-CReSA46 (KX249751)
PRRSFLEX BI (KT988004)
ESP-2014-CReSA70 (KX249752)
ESP-2013-CReSA261 (KX249756)
ESP-1992-Cresa3249 (JF276433)
ESP-2005-Cresa3256 (JF276432)
ESP-2014-CReSA100 (KX249753)
ESP-2013-CReSA184 (KX249754)
ESP-2014-CReSA38 (KX249750)
ESP-2014-CReSA17 (KX249749)
ESP-2013-CReSA3 (KX249748)
96V198 (LQ787782)
HKG-2007-HKEU16 (EU076704)
HKG-2004-HK5 (KF287130)
HKG-2004-HK10 (KF287131)
HKG-2003-HK3 (KF287129)
HKG-2004-HK8 (KF287128)
ESP-2005-Cresa3262 (JF276431)
HUN-2016-HU19483_2016 (MH463458)
BEL-2013-13V91 (KT159248)
HUN-2016-HU19401_2016 (MH463457)
ITA-2014-PR40_2014 (MF346695)
KOR-2011-D40 (KY434184)
KOR-2007-E38 (KT033457)
KOR-2007-KNU-07 (FJ349261)
GER-2013-613 (KT326148)
AUT-2013-883 (KT326148)
GER-2009-GER09-613 (KT344816)
KOR-2016-CBNU0495 (KY434183)
AUT-2014-440 (KT334375)
CHE-2012-IVI-1173 (KX622783)
HUN-2016-HU24924_2016 (MH463459)
HUN-2016-HU18755_2016 (MH463455)
HUN-2016-HU18861_2016 (MH463456)
CHN-2018-180900-5 (MK303390)
RUS-2013-WestSib13 (KX668221)
Lena (JF802085)
BLR-2010-SU1-Bel (KP889243)
VR2332 (PRU87392)
0.04

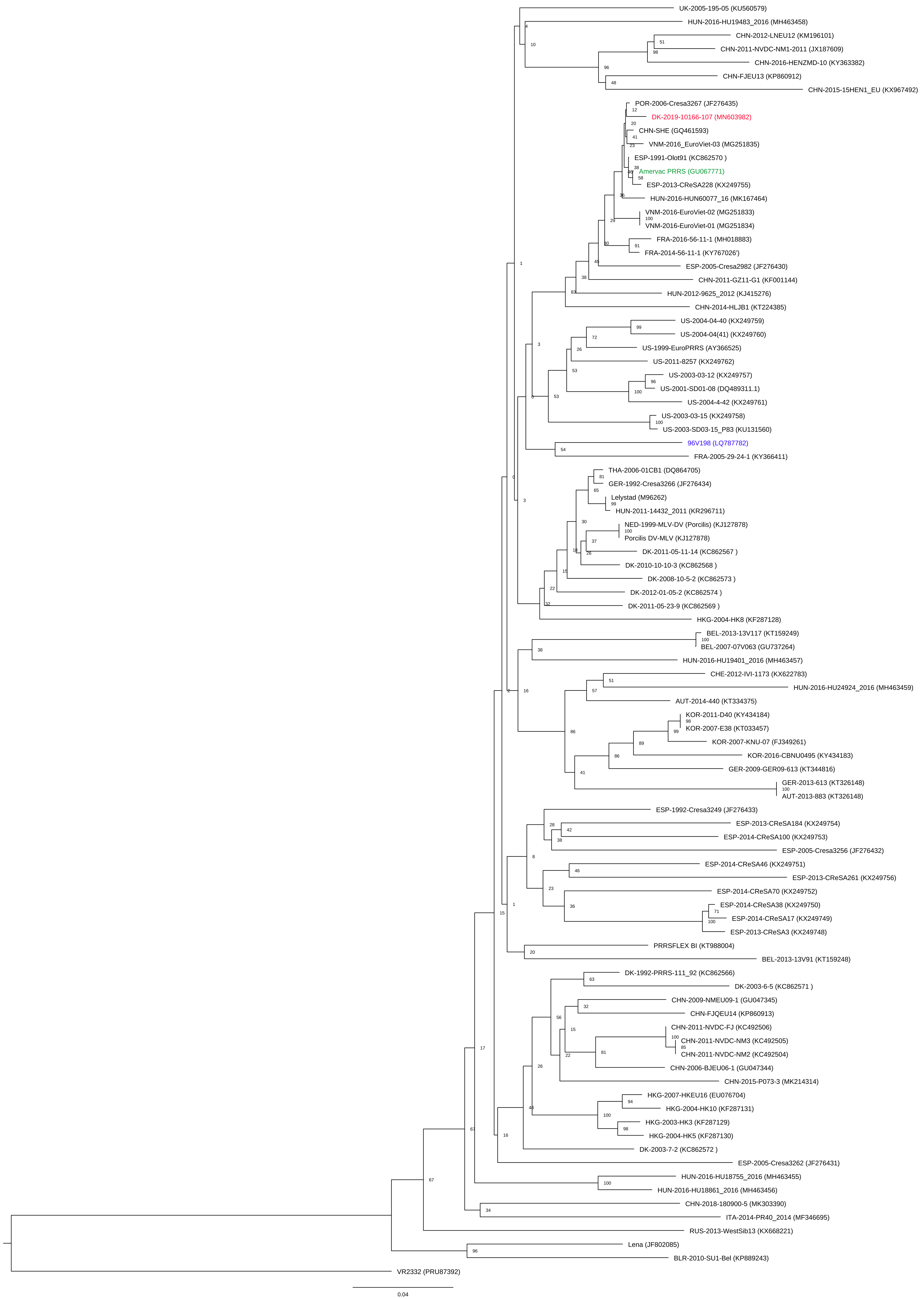
UK-2005-195-05 (KU560579)
HUN-2016-HU19483_2016 (MH463458)
CHN-2012-LNEU12 (KM196101)
CHN-2011-NVDC-NM1-2011 (JX187609)
CHN-2016-HENZMD-10 (KY363382)
CHN-FJEU13 (KP860912)
CHN-2015-15HEN1_EU (KX967492)
POR-2006-Cresa3267 (JF276435)
DK-2019-10166-107 (MN603982)
CHN-SHE (GQ461593)
VNM-2016_EuroViet-03 (MG251835)
ESP-1991-Olot91 (KC862570)
Amervac PRRS (GU067771)
ESP-2013-CReSA228 (KX249755)
HUN-2016-HUN60077_16 (MK167464)
VNM-2016-EuroViet-02 (MG251833)
VNM-2016-EuroViet-01 (MG251834)
FRA-2016-56-11-1 (MH018883)
FRA-2014-56-11-1 (KY767026')
ESP-2005-Cresa2982 (JF276430)
CHN-2011-GZ11-G1 (KF001144)
HUN-2012-9625_2012 (KJ415276)
CHN-2014-HLJB1 (KT224385)
US-2004-04-40 (KX249759)
US-2004-04(41) (KX249760)
US-1999-EuroPRRS (AY366525)
US-2011-8257 (KX249762)
US-2003-03-12 (KX249757)
US-2001-SD01-08 (DQ489311.1)
US-2004-4-42 (KX249761)
US-2003-03-15 (KX249758)
US-2003-SD03-15_P83 (KU131560)
96V198 (LQ787782)
FRA-2005-29-24-1 (KY366411)
THA-2006-01CB1 (DQ864705)
GER-1992-Cresa3266 (JF276434)
Lelystad (M96262)
HUN-2011-14432_2011 (KR296711)
NED-1999-MLV-DV (Porcilis) (KJ127878)
Porcilis DV-MLV (KJ127878)
DK-2011-05-11-14 (KC862567)
DK-2010-10-10-3 (KC862568)
DK-2008-10-5-2 (KC862573)
DK-2012-01-05-2 (KC862574)
DK-2011-05-23-9 (KC862569)
HKG-2004-HK8 (KF287128)
BEL-2013-13V117 (KT159249)
BEL-2007-07V063 (GU737264)
HUN-2016-HU19401_2016 (MH463457)
CHE-2012-IVI-1173 (KX622783)
HUN-2016-HU24924_2016 (MH463459)
AUT-2014-440 (KT334375)
KOR-2011-D40 (KY434184)
KOR-2007-E38 (KT033457)
KOR-2007-KNU-07 (FJ349261)
KOR-2016-CBNU0495 (KY434183)
GER-2009-GER09-613 (KT344816)
GER-2013-613 (KT326148)
AUT-2013-883 (KT326148)
ESP-1992-Cresa3249 (JF276433)
ESP-2013-CReSA184 (KX249754)
ESP-2014-CReSA100 (KX249753)
ESP-2005-Cresa3256 (JF276432)
ESP-2014-CReSA46 (KX249751)
ESP-2013-CReSA261 (KX249756)
ESP-2014-CReSA70 (KX249752)
ESP-2014-CReSA38 (KX249750)
ESP-2014-CReSA17 (KX249749)
ESP-2013-CReSA3 (KX249748)
PRRSFLEX BI (KT988004)
BEL-2013-13V91 (KT159248)
DK-1992-PRRS-111_92 (KC862566)
DK-2003-6-5 (KC862571)
CHN-2009-NMEU09-1 (GU047345)
CHN-FJQEU14 (KP860913)
CHN-2011-NVDC-FJ (KC492506)
CHN-2011-NVDC-NM3 (KC492505)
CHN-2011-NVDC-NM2 (KC492504)
CHN-2006-BJEU06-1 (GU047344)
CHN-2015-P073-3 (MK214314)
HKG-2007-HKEU16 (EU076704)
HKG-2004-HK10 (KF287131)
HKG-2003-HK3 (KF287129)
HKG-2004-HK5 (KF287130)
DK-2003-7-2 (KC862572)
ESP-2005-Cresa3262 (JF276431)
HUN-2016-HU18755_2016 (MH463455)
HUN-2016-HU18861_2016 (MH463456)
CHN-2018-180900-5 (MK303390)
ITA-2014-PR40_2014 (MF346695)
RUS-2013-WestSib13 (KX668221)
Lena (JF802085)
BLR-2010-SU1-Bel (KP889243)
VR2332 (PRU87392)
0.04

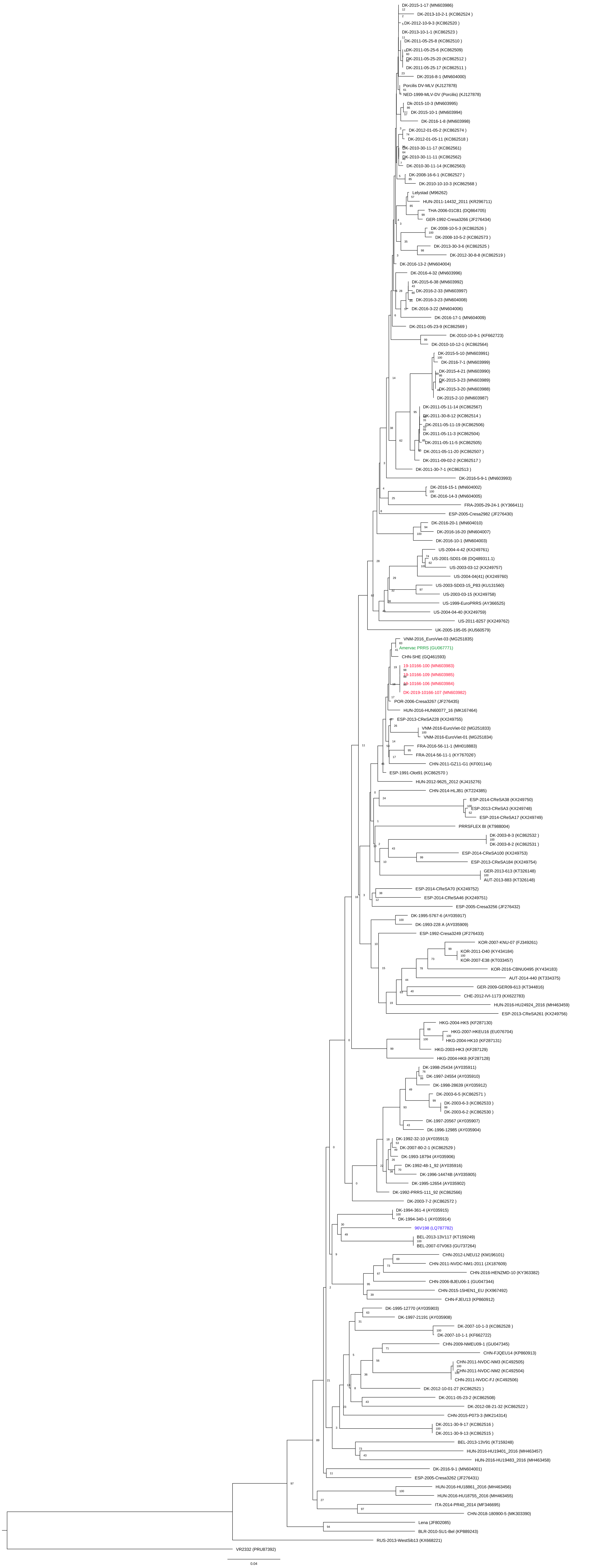

DK-2015-1-17 (MN603986)
DK-2013-10-2-1 (KC862524)
DK-2012-10-9-3 (KC862520)
DK-2013-10-1-1 (KC862523)
DK-2011-05-25-8 (KC862510)
DK-2011-05-25-6 (KC862509)
DK-2011-05-25-20 (KC862512)
DK-2011-05-25-17 (KC862511)
DK-2016-8-1 (MN604000)
Porcilis DV-MLV (KJ127878)
NED-1999-MLV-DV (Porcilis) (KJ127878)
DK-2015-10-3 (MN603995)
DK-2015-10-1 (MN603994)
DK-2016-1-8 (MN603998)
DK-2012-01-05-2 (KC862574)
DK-2012-01-05-11 (KC862518)
DK-2010-30-11-17 (KC862561)
DK-2010-30-11-11 (KC862562)
DK-2010-30-11-14 (KC862563)
DK-2008-16-6-1 (KC862527)
DK-2010-10-10-3 (KC862568)
Lelystad (M96262)
HUN-2011-14432_2011 (KR296711)
THA-2006-01CB1 (DQ864705)
GER-1992-Cresa3266 (JF276434)
DK-2008-10-5-3 (KC862526)
DK-2008-10-5-2 (KC862573)
DK-2013-30-3-6 (KC862525)
DK-2012-30-8-8 (KC862519)
DK-2016-13-2 (MN604004)
DK-2016-4-32 (MN603996)
DK-2015-6-38 (MN603992)
DK-2016-2-33 (MN603997)
DK-2016-3-23 (MN604008)
DK-2016-3-22 (MN604006)
DK-2016-17-1 (MN604009)
DK-2011-05-23-9 (KC862569)
DK-2010-10-9-1 (KF662723)
DK-2010-10-12-1 (KC862564)
DK-2015-5-10 (MN603991)
DK-2016-7-1 (MN603999)
DK-2015-4-21 (MN603990)
DK-2015-3-23 (MN603989)
DK-2015-3-20 (MN603988)
DK-2015-2-10 (MN603987)
DK-2011-05-11-14 (KC862567)
DK-2011-30-8-12 (KC862514)
DK-2011-05-11-19 (KC862506)
DK-2011-05-11-3 (KC862504)
DK-2011-05-11-5 (KC862505)
DK-2011-05-11-20 (KC862507)
DK-2011-09-02-2 (KC862517)
DK-2011-30-7-1 (KC862513)
DK-2016-5-9-1 (MN603993)
DK-2016-15-1 (MN604002)
DK-2016-14-3 (MN604005)
FRA-2005-29-24-1 (KY366411)
ESP-2005-Cresa2982 (JF276430)
DK-2016-20-1 (MN604010)
DK-2016-16-20 (MN604007)
DK-2016-10-1 (MN604003)
US-2004-4-42 (KX249761)
US-2001-SD01-08 (DQ489311.1)
US-2003-03-12 (KX249757)
US-2004-04(41) (KX249760)
US-2003-SD03-15_P83 (KU131560)
US-2003-03-15 (KX249758)
US-1999-EuroPRRS (AY366525)
US-2004-04-40 (KX249759)
US-2011-8257 (KX249762)
UK-2005-195-05 (KU560579)
VNM-2016_EuroViet-03 (MG251835)
Amervac PRRS (GU067771)
CHN-SHE (GQ461593)
19-10166-100 (MN603983)
19-10166-109 (MN603985)
19-10166-106 (MN603984)
DK-2019-10166-107 (MN603982)
POR-2006-Cresa3267 (JF276435)
HUN-2016-HUN60077_16 (MK167464)
ESP-2013-CReSA228 (KX249755)
VNM-2016-EuroViet-02 (MG251833)
VNM-2016-EuroViet-01 (MG251834)
FRA-2016-56-11-1 (MH018883)
FRA-2014-56-11-1 (KY767026)
CHN-2011-GZ11-G1 (KF001144)
ESP-1991-Olot91 (KC862570)
HUN-2012-9625_2012 (KJ415276)
CHN-2014-HLJB1 (KT224385)
ESP-2014-CReSA38 (KX249750)
ESP-2013-CReSA3 (KX249748)
ESP-2014-CReSA17 (KX249749)
PRRSFLEX BI (KT988004)
DK-2003-8-3 (KC862532)
DK-2003-8-2 (KC862531)
ESP-2014-CReSA100 (KX249753)
ESP-2013-CReSA184 (KX249754)
GER-2013-613 (KT326148)
AUT-2013-883 (KT326148)
ESP-2014-CReSA70 (KX249752)
ESP-2014-CReSA46 (KX249751)
ESP-2005-Cresa3256 (JF276432)
DK-1995-5767-6 (AY035917)
DK-1993-228 A (AY035909)
ESP-1992-Cresa3249 (JF276433)
KOR-2007-KNU-07 (FJ349261)
KOR-2011-D40 (KY434184)
KOR-2007-E38 (KT033457)
KOR-2016-CBNU0495 (KY434183)
AUT-2014-440 (KT334375)
GER-2009-GER09-613 (KT344816)
CHE-2012-IVI-1173 (KX622783)
HUN-2016-HU24924_2016 (MH463459)
ESP-2013-CReSA261 (KX249756)
HKG-2004-HK5 (KF287130)
HKG-2007-HKEU16 (EU076704)
HKG-2004-HK10 (KF287131)
HKG-2003-HK3 (KF287129)
HKG-2004-HK8 (KF287128)
DK-1998-25434 (AY035911)
DK-1997-24554 (AY035910)
DK-1998-28639 (AY035912)
DK-2003-6-5 (KC862571)
DK-2003-6-3 (KC862533)
DK-2003-6-2 (KC862530)
DK-1997-20567 (AY035907)
DK-1996-12985 (AY035904)
DK-1992-32-10 (AY035913)
DK-2007-80-2-1 (KC862529)
DK-1993-18794 (AY035906)
DK-1992-48-1_92 (AY035916)
DK-1996-14474B (AY035905)
DK-1995-12654 (AY035902)
DK-1992-PRRS-111_92 (KC862566)
DK-2003-7-2 (KC862572)
DK-1994-361-4 (AY035915)
DK-1994-340-1 (AY035914)
96V198 (LQ787782)
BEL-2013-13V117 (KT159249)
BEL-2007-07V063 (GU737264)
CHN-2012-LNEU12 (KM196101)
CHN-2011-NVDC-NM1-2011 (JX187609)
CHN-2016-HENZMD-10 (KY363382)
CHN-2006-BJEU06-1 (GU047344)
CHN-2015-15HEN1_EU (KX967492)
CHN-FJEU13 (KP860912)
DK-1995-12770 (AY035903)
DK-1997-21191 (AY035908)
DK-2007-10-1-3 (KC862528)
DK-2007-10-1-1 (KF662722)
CHN-2009-NMEU09-1 (GU047345)
CHN-FJQEU14 (KP860913)
CHN-2011-NVDC-NM3 (KC492505)
CHN-2011-NVDC-NM2 (KC492504)
CHN-2011-NVDC-FJ (KC492506)
DK-2012-10-01-27 (KC862521)
DK-2011-05-23-2 (KC862508)
DK-2012-08-21-32 (KC862522)
CHN-2015-P073-3 (MK214314)
DK-2011-30-9-17 (KC862516)
DK-2011-30-9-13 (KC862515)
BEL-2013-13V91 (KT159248)
HUN-2016-HU19401_2016 (MH463457)
HUN-2016-HU19483_2016 (MH463458)
DK-2016-9-1 (MN604001)
ESP-2005-Cresa3262 (JF276431)
HUN-2016-HU18861_2016 (MH463456)
HUN-2016-HU18755_2016 (MH463455)
ITA-2014-PR40_2014 (MF346695)
CHN-2018-180900-5 (MK303390)
Lena (JF802085)
BLR-2010-SU1-Bel (KP889243)
RUS-2013-WestSib13 (KX668221)
VR2332 (PRU87392)
0.04

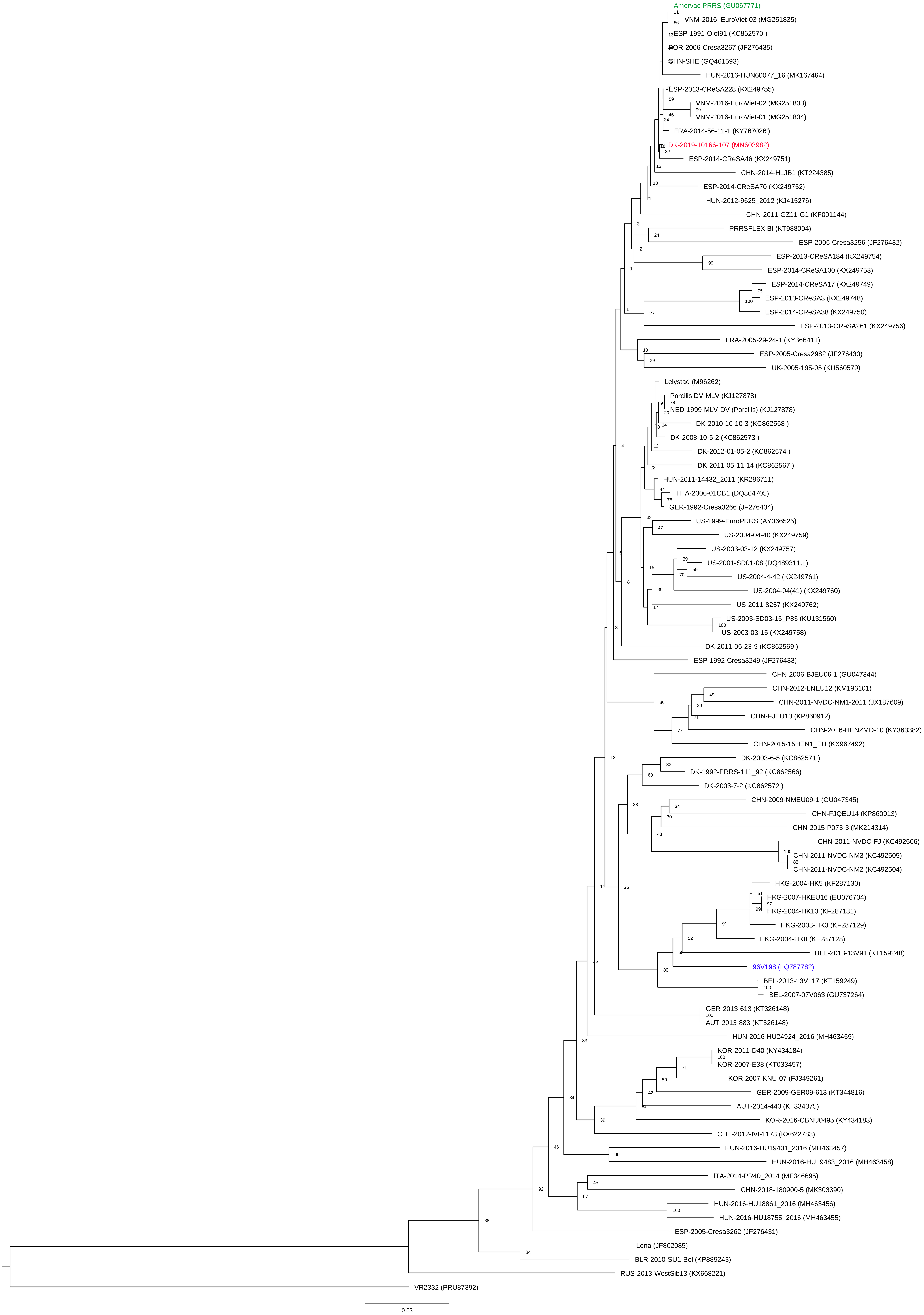

Amervac PRRS (GU067771)
VNM-2016_EuroViet-03 (MG251835)
ESP-1991-Olot91 (KC862570)
POR-2006-Cresa3267 (JF276435)
CHN-SHE (GQ461593)
HUN-2016-HUN60077_16 (MK167464)
ESP-2013-CReSA228 (KX249755)
VNM-2016-EuroViet-02 (MG251833)
VNM-2016-EuroViet-01 (MG251834)
FRA-2014-56-11-1 (KY767026')
DK-2019-10166-107 (MN603982)
ESP-2014-CReSA46 (KX249751)
CHN-2014-HLJB1 (KT224385)
ESP-2014-CReSA70 (KX249752)
HUN-2012-9625_2012 (KJ415276)
CHN-2011-GZ11-G1 (KF001144)
PRRSFLEX BI (KT988004)
ESP-2005-Cresa3256 (JF276432)
ESP-2013-CReSA184 (KX249754)
ESP-2014-CReSA100 (KX249753)
ESP-2014-CReSA17 (KX249749)
ESP-2013-CReSA3 (KX249748)
ESP-2014-CReSA38 (KX249750)
ESP-2013-CReSA261 (KX249756)
FRA-2005-29-24-1 (KY366411)
ESP-2005-Cresa2982 (JF276430)
UK-2005-195-05 (KU560579)
Lelystad (M96262)
Porcilis DV-MLV (KJ127878)
NED-1999-MLV-DV (Porcilis) (KJ127878)
DK-2010-10-10-3 (KC862568)
DK-2008-10-5-2 (KC862573)
DK-2012-01-05-2 (KC862574)
DK-2011-05-11-14 (KC862567)
HUN-2011-14432_2011 (KR296711)
THA-2006-01CB1 (DQ864705)
GER-1992-Cresa3266 (JF276434)
US-1999-EuroPRRS (AY366525)
US-2004-04-40 (KX249759)
US-2003-03-12 (KX249757)
US-2001-SD01-08 (DQ489311.1)
US-2004-4-42 (KX249761)
US-2004-04(41) (KX249760)
US-2011-8257 (KX249762)
US-2003-SD03-15_P83 (KU131560)
US-2003-03-15 (KX249758)
DK-2011-05-23-9 (KC862569)
ESP-1992-Cresa3249 (JF276433)
CHN-2006-BJEU06-1 (GU047344)
CHN-2012-LNEU12 (KM196101)
CHN-2011-NVDC-NM1-2011 (JX187609)
CHN-FJEU13 (KP860912)
CHN-2016-HENZMD-10 (KY363382)
CHN-2015-15HEN1_EU (KX967492)
DK-2003-6-5 (KC862571)
DK-1992-PRRS-111_92 (KC862566)
DK-2003-7-2 (KC862572)
CHN-2009-NMEU09-1 (GU047345)
CHN-FJQEU14 (KP860913)
CHN-2015-P073-3 (MK214314)
CHN-2011-NVDC-FJ (KC492506)
CHN-2011-NVDC-NM3 (KC492505)
CHN-2011-NVDC-NM2 (KC492504)
HKG-2004-HK5 (KF287130)
HKG-2007-HKEU16 (EU076704)
HKG-2004-HK10 (KF287131)
HKG-2003-HK3 (KF287129)
HKG-2004-HK8 (KF287128)
BEL-2013-13V91 (KT159248)
96V198 (LQ787782)
BEL-2013-13V117 (KT159249)
BEL-2007-07V063 (GU737264)
GER-2013-613 (KT326148)
AUT-2013-883 (KT326148)
HUN-2016-HU24924_2016 (MH463459)
KOR-2011-D40 (KY434184)
KOR-2007-E38 (KT033457)
KOR-2007-KNU-07 (FJ349261)
GER-2009-GER09-613 (KT344816)
AUT-2014-440 (KT334375)
KOR-2016-CBNU0495 (KY434183)
CHE-2012-IVI-1173 (KX622783)
HUN-2016-HU19401_2016 (MH463457)
HUN-2016-HU19483_2016 (MH463458)
ITA-2014-PR40_2014 (MF346695)
CHN-2018-180900-5 (MK303390)
HUN-2016-HU18861_2016 (MH463456)
HUN-2016-HU18755_2016 (MH463455)
ESP-2005-Cresa3262 (JF276431)
Lena (JF802085)
BLR-2010-SU1-Bel (KP889243)
RUS-2013-WestSib13 (KX668221)
VR2332 (PRU87392)
0.03

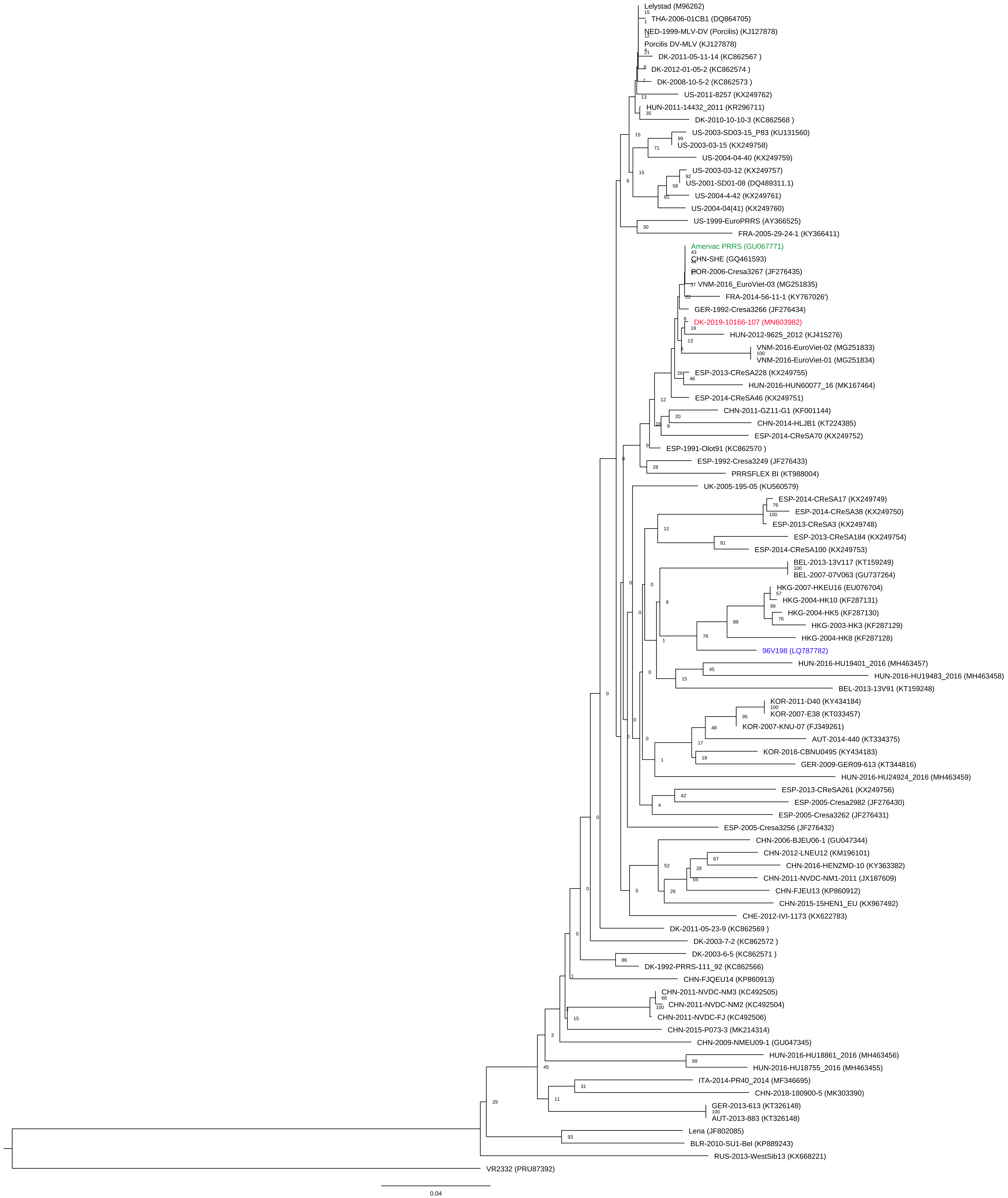
Lelystad (M96262)
THA-2006-01CB1 (DQ864705)
NED-1999-MLV-DV (Porcilis) (KJ127878)
Porcilis DV-MLV (KJ127878)
DK-2011-05-11-14 (KC862567)
DK-2012-01-05-2 (KC862574)
DK-2008-10-5-2 (KC862573)
US-2011-8257 (KX249762)
HUN-2011-14432_2011 (KR296711)
DK-2010-10-10-3 (KC862568)
US-2003-SD03-15_P83 (KU131560)
US-2003-03-15 (KX249758)
US-2004-04-40 (KX249759)
US-2003-03-12 (KX249757)
US-2001-SD01-08 (DQ489311.1)
US-2004-4-42 (KX249761)
US-2004-04(41) (KX249760)
US-1999-EuroPRRS (AY366525)
FRA-2005-29-24-1 (KY366411)
Amervac PRRS (GU067771)
CHN-SHE (GQ461593)
POR-2006-Cresa3267 (JF276435)
VNM-2016_EuroViet-03 (MG251835)
FRA-2014-56-11-1 (KY767026')
GER-1992-Cresa3266 (JF276434)
DK-2019-10166-107 (MN603982)
HUN-2012-9625_2012 (KJ415276)
VNM-2016-EuroViet-02 (MG251833)
VNM-2016-EuroViet-01 (MG251834)
ESP-2013-CReSA228 (KX249755)
HUN-2016-HUN60077_16 (MK167464)
ESP-2014-CReSA46 (KX249751)
CHN-2011-GZ11-G1 (KF001144)
CHN-2014-HLJB1 (KT224385)
ESP-2014-CReSA70 (KX249752)
ESP-1991-Olot91 (KC862570)
ESP-1992-Cresa3249 (JF276433)
PRRSFLEX BI (KT988004)
UK-2005-195-05 (KU560579)
ESP-2014-CReSA17 (KX249749)
ESP-2014-CReSA38 (KX249750)
ESP-2013-CReSA3 (KX249748)
ESP-2013-CReSA184 (KX249754)
ESP-2014-CReSA100 (KX249753)
BEL-2013-13V117 (KT159249)
BEL-2007-07V063 (GU737264)
HKG-2007-HKEU16 (EU076704)
HKG-2004-HK10 (KF287131)
HKG-2004-HK5 (KF287130)
HKG-2003-HK3 (KF287129)
HKG-2004-HK8 (KF287128)
96V198 (LQ787782)
HUN-2016-HU19401_2016 (MH463457)
HUN-2016-HU19483_2016 (MH463458)
BEL-2013-13V91 (KT159248)
KOR-2011-D40 (KY434184)
KOR-2007-E38 (KT033457)
KOR-2007-KNU-07 (FJ349261)
AUT-2014-440 (KT334375)
KOR-2016-CBNU0495 (KY434183)
GER-2009-GER09-613 (KT344816)
HUN-2016-HU24924_2016 (MH463459)
ESP-2013-CReSA261 (KX249756)
ESP-2005-Cresa2982 (JF276430)
ESP-2005-Cresa3262 (JF276431)
ESP-2005-Cresa3256 (JF276432)
CHN-2006-BJEU06-1 (GU047344)
CHN-2012-LNEU12 (KM196101)
CHN-2016-HENZMD-10 (KY363382)
CHN-2011-NVDC-NM1-2011 (JX187609)
CHN-FJEU13 (KP860912)
CHN-2015-15HEN1_EU (KX967492)
CHE-2012-IVI-1173 (KX622783)
DK-2011-05-23-9 (KC862569)
DK-2003-7-2 (KC862572)
DK-2003-6-5 (KC862571)
DK-1992-PRRS-111_92 (KC862566)
CHN-FJQEU14 (KP860913)
CHN-2011-NVDC-NM3 (KC492505)
CHN-2011-NVDC-NM2 (KC492504)
CHN-2011-NVDC-FJ (KC492506)
CHN-2015-P073-3 (MK214314)
CHN-2009-NMEU09-1 (GU047345)
HUN-2016-HU18861_2016 (MH463456)
HUN-2016-HU18755_2016 (MH463455)
ITA-2014-PR40_2014 (MF346695)
CHN-2018-180900-5 (MK303390)
GER-2013-613 (KT326148)
AUT-2013-883 (KT326148)
Lena (JF802085)
BLR-2010-SU1-Bel (KP889243)
RUS-2013-WestSib13 (KX668221)
VR2332 (PRU87392)
0.04

Supplementary Figure 2

Supplementary Figure 2: Phylogenetic trees of ORF3. (a) downstream (nucleotide 1-201) and (b) upstream (nucleotide 202-798) of putative breakpoint. The phylogenetic trees were constructed using the Neighbor Joining method with Jukes-Cantor as the nucleotide distance measure and bootstrap analysis with 1000 replicates. PRRSV-2 VR2332 (PRU87392) was used as outgroup. Trees were drawn using FigTree v.1.4.3.

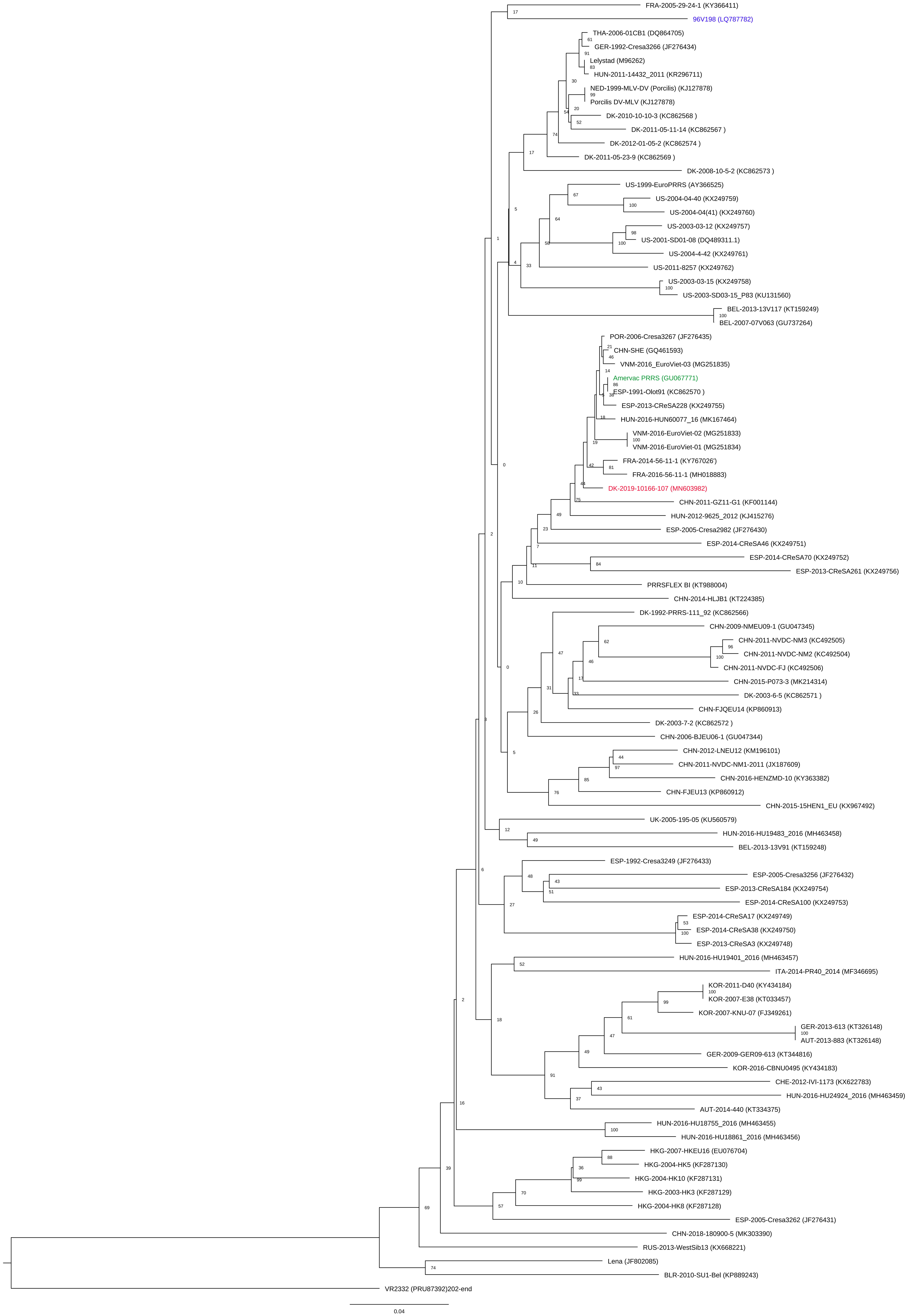
FRA-2005-29-24-1 (KY366411)
96V198 (LQ787782)
THA-2006-01CB1 (DQ864705)
GER-1992-Cresa3266 (JF276434)
Lelystad (M96262)
HUN-2011-14432_2011 (KR296711)
NED-1999-MLV-DV (Porcilis) (KJ127878)
Porcilis DV-MLV (KJ127878)
DK-2010-10-10-3 (KC862568)
DK-2011-05-11-14 (KC862567)
DK-2012-01-05-2 (KC862574)
DK-2011-05-23-9 (KC862569)
DK-2008-10-5-2 (KC862573)
US-1999-EuroPRRS (AY366525)
US-2004-04-40 (KX249759)
US-2004-04(41) (KX249760)
US-2003-03-12 (KX249757)
US-2001-SD01-08 (DQ489311.1)
US-2004-4-42 (KX249761)
US-2011-8257 (KX249762)
US-2003-03-15 (KX249758)
US-2003-SD03-15_P83 (KU131560)
BEL-2013-13V117 (KT159249)
BEL-2007-07V063 (GU737264)
POR-2006-Cresa3267 (JF276435)
CHN-SHE (GQ461593)
VNM-2016_EuroViet-03 (MG251835)
Amervac PRRS (GU067771)
ESP-1991-Olot91 (KC862570)
ESP-2013-CReSA228 (KX249755)
HUN-2016-HUN60077_16 (MK167464)
VNM-2016-EuroViet-02 (MG251833)
VNM-2016-EuroViet-01 (MG251834)
FRA-2014-56-11-1 (KY767026')
FRA-2016-56-11-1 (MH018883)
DK-2019-10166-107 (MN603982)
CHN-2011-GZ11-G1 (KF001144)
HUN-2012-9625_2012 (KJ415276)
ESP-2005-Cresa2982 (JF276430)
ESP-2014-CReSA46 (KX249751)
ESP-2014-CReSA70 (KX249752)
ESP-2013-CReSA261 (KX249756)
PRRSFLEX BI (KT988004)
CHN-2014-HLJB1 (KT224385)
DK-1992-PRRS-111_92 (KC862566)
CHN-2009-NMEU09-1 (GU047345)
CHN-2011-NVDC-NM3 (KC492505)
CHN-2011-NVDC-NM2 (KC492504)
CHN-2011-NVDC-FJ (KC492506)
CHN-2015-P073-3 (MK214314)
DK-2003-6-5 (KC862571)
CHN-FJQEU14 (KP860913)
DK-2003-7-2 (KC862572)
CHN-2006-BJEU06-1 (GU047344)
CHN-2012-LNEU12 (KM196101)
CHN-2011-NVDC-NM1-2011 (JX187609)
CHN-2016-HENZMD-10 (KY363382)
CHN-FJEU13 (KP860912)
CHN-2015-15HEN1_EU (KX967492)
UK-2005-195-05 (KU560579)
HUN-2016-HU19483_2016 (MH463458)
BEL-2013-13V91 (KT159248)
ESP-1992-Cresa3249 (JF276433)
ESP-2005-Cresa3256 (JF276432)
ESP-2013-CReSA184 (KX249754)
ESP-2014-CReSA100 (KX249753)
ESP-2014-CReSA17 (KX249749)
ESP-2014-CReSA38 (KX249750)
ESP-2013-CReSA3 (KX249748)
HUN-2016-HU19401_2016 (MH463457)
ITA-2014-PR40_2014 (MF346695)
KOR-2011-D40 (KY434184)
KOR-2007-E38 (KT033457)
KOR-2007-KNU-07 (FJ349261)
GER-2013-613 (KT326148)
AUT-2013-883 (KT326148)
GER-2009-GER09-613 (KT344816)
KOR-2016-CBNU0495 (KY434183)
CHE-2012-IVI-1173 (KX622783)
HUN-2016-HU24924_2016 (MH463459)
AUT-2014-440 (KT334375)
HUN-2016-HU18755_2016 (MH463455)
HUN-2016-HU18861_2016 (MH463456)
HKG-2007-HKEU16 (EU076704)
HKG-2004-HK5 (KF287130)
HKG-2004-HK10 (KF287131)
HKG-2003-HK3 (KF287129)
HKG-2004-HK8 (KF287128)
ESP-2005-Cresa3262 (JF276431)
CHN-2018-180900-5 (MK303390)
RUS-2013-WestSib13 (KX668221)
Lena (JF802085)
BLR-2010-SU1-Bel (KP889243)
VR2332 (PRU87392)202-end
0.04

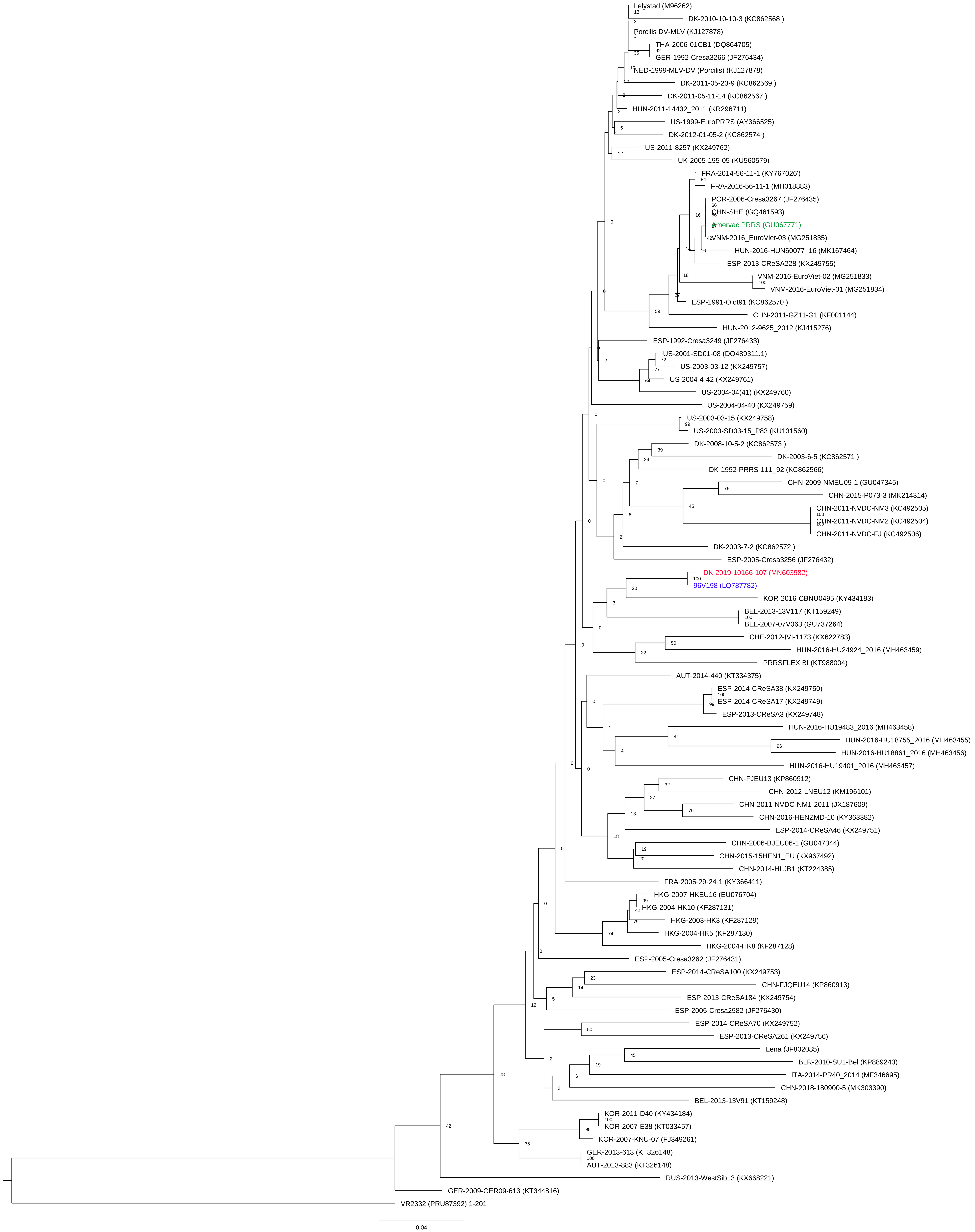
Lelystad (M96262)
DK-2010-10-10-3 (KC862568)
Porcilis DV-MLV (KJ127878)
THA-2006-01CB1 (DQ864705)
GER-1992-Cresa3266 (JF276434)
NED-1999-MLV-DV (Porcilis) (KJ127878)
DK-2011-05-23-9 (KC862569)
DK-2011-05-11-14 (KC862567)
HUN-2011-14432_2011 (KR296711)
US-1999-EuroPRRS (AY366525)
DK-2012-01-05-2 (KC862574)
US-2011-8257 (KX249762)
UK-2005-195-05 (KU560579)
FRA-2014-56-11-1 (KY767026')
FRA-2016-56-11-1 (MH018883)
POR-2006-Cresa3267 (JF276435)
CHN-SHE (GQ461593)
Amervac PRRS (GU067771)
VNM-2016_EuroViet-03 (MG251835)
HUN-2016-HUN60077_16 (MK167464)
ESP-2013-CReSA228 (KX249755)
VNM-2016-EuroViet-02 (MG251833)
VNM-2016-EuroViet-01 (MG251834)
ESP-1991-Olot91 (KC862570)
CHN-2011-GZ11-G1 (KF001144)
HUN-2012-9625_2012 (KJ415276)
ESP-1992-Cresa3249 (JF276433)
US-2001-SD01-08 (DQ489311.1)
US-2003-03-12 (KX249757)
US-2004-4-42 (KX249761)
US-2004-04(41) (KX249760)
US-2004-04-40 (KX249759)
US-2003-03-15 (KX249758)
US-2003-SD03-15_P83 (KU131560)
DK-2008-10-5-2 (KC862573)
DK-2003-6-5 (KC862571)
DK-1992-PRRS-111_92 (KC862566)
CHN-2009-NMEU09-1 (GU047345)
CHN-2015-P073-3 (MK214314)
CHN-2011-NVDC-NM3 (KC492505)
CHN-2011-NVDC-NM2 (KC492504)
CHN-2011-NVDC-FJ (KC492506)
DK-2003-7-2 (KC862572)
ESP-2005-Cresa3256 (JF276432)
DK-2019-10166-107 (MN603982)
96V198 (LQ787782)
KOR-2016-CBNU0495 (KY434183)
BEL-2013-13V117 (KT159249)
BEL-2007-07V063 (GU737264)
CHE-2012-IVI-1173 (KX622783)
HUN-2016-HU24924_2016 (MH463459)
PRRSFLEX BI (KT988004)
AUT-2014-440 (KT334375)
ESP-2014-CReSA38 (KX249750)
ESP-2014-CReSA17 (KX249749)
ESP-2013-CReSA3 (KX249748)
HUN-2016-HU19483_2016 (MH463458)
HUN-2016-HU18755_2016 (MH463455)
HUN-2016-HU18861_2016 (MH463456)
HUN-2016-HU19401_2016 (MH463457)
CHN-FJEU13 (KP860912)
CHN-2012-LNEU12 (KM196101)
CHN-2011-NVDC-NM1-2011 (JX187609)
CHN-2016-HENZMD-10 (KY363382)
ESP-2014-CReSA46 (KX249751)
CHN-2006-BJEU06-1 (GU047344)
CHN-2015-15HEN1_EU (KX967492)
CHN-2014-HLJB1 (KT224385)
FRA-2005-29-24-1 (KY366411)
HKG-2007-HKEU16 (EU076704)
HKG-2004-HK10 (KF287131)
HKG-2003-HK3 (KF287129)
HKG-2004-HK5 (KF287130)
HKG-2004-HK8 (KF287128)
ESP-2005-Cresa3262 (JF276431)
ESP-2014-CReSA100 (KX249753)
CHN-FJQEU14 (KP860913)
ESP-2013-CReSA184 (KX249754)
ESP-2005-Cresa2982 (JF276430)
ESP-2014-CReSA70 (KX249752)
ESP-2013-CReSA261 (KX249756)
Lena (JF802085)
BLR-2010-SU1-Bel (KP889243)
ITA-2014-PR40_2014 (MF346695)
CHN-2018-180900-5 (MK303390)
BEL-2013-13V91 (KT159248)
KOR-2011-D40 (KY434184)
KOR-2007-E38 (KT033457)
KOR-2007-KNU-07 (FJ349261)
GER-2013-613 (KT326148)
AUT-2013-883 (KT326148)
RUS-2013-WestSib13 (KX668221)
GER-2009-GER09-613 (KT344816)
VR2332 (PRU87392) 1-201
0.04
